# Electrical Properties Tomography: A Methodological Review

**DOI:** 10.3390/diagnostics11020176

**Published:** 2021-01-26

**Authors:** Reijer Leijsen, Wyger Brink, Cornelis van den Berg, Andrew Webb, Rob Remis

**Affiliations:** 1 Department of Radiology, C.J. Gorter Center for High Field MRI, Leiden University Medical Center, 2333ZA Leiden, The Netherlands; R.L.Leijsen@lumc.nl (R.L.); W.M.Brink@lumc.nl (W.B.); A.Webb@lumc.nl (A.W.); 2 Computational Imaging Group for MRI Diagnostics and Therapy, Centre for Image Sciences, University Medical Centre Utrecht, 3508GA Utrecht, The Netherlands; C.A.T.vandenBerg@umcutrecht.nl; 3 Circuits and Systems Group, Faculty of Electrical Engineering, Mathematics and Computes Science, Delft University of Technology, 2628CD Delft, The Netherlands

**Keywords:** electrical properties tomography (EPT), conductivity, permittivity, inversion, magnetic resonance imaging (MRI)

## Abstract

Electrical properties tomography (EPT) is an imaging method that uses a magnetic resonance (MR) system to non-invasively determine the spatial distribution of the conductivity and permittivity of the imaged object. This manuscript starts by providing clear definitions about the data required for, and acquired in, EPT, followed by comprehensively formulating the physical equations underlying a large number of analytical EPT techniques. This thorough mathematical overview of EPT harmonizes several EPT techniques in a single type of formulation and gives insight into how they act on the data and what their data requirements are. Furthermore, the review describes machine learning-based algorithms. Matlab code of several differential and iterative integral methods is available upon request.

## 1. Introduction

The electrical properties (EPs; conductivity σ and permittivity ε) of tissue have the potential to be used as biomarkers in many clinical applications. Tissue EPs depend on the tissue structure and composition. The conductivity varies largely as a function of fluid volumes and ionic concentrations, while the permittivity is largely influenced by the cellular membrane extent [1]. Cancer causes local changes of EPs relative to healthy tissues. The EPs of benign tissue compared to tumors are significantly different and have been reported to offer advantages in separating them from each other [2,3,4]. Similarly, the conductivity in cerebral ischemia is significantly decreased [5,6]. Conductivity measurements can therefore be helpful for better characterization of brain tumors [7,8], but they have also shown promising results for pelvic tumors [9], breast cancer [10] and ischemic stroke [11,12]. Knowledge of the EPs additionally allows for the calculation of the electromagnetic (EM) fields inside tissue. This makes them interesting for a wide range of clinical applications, such as electroencephalography (EEG) and electrocardiography (ECG) measurements to accurately localize internal electrical activities, deep brain stimulation to mitigate Parkinson’s disease symptoms, radio frequency (RF) ablation to remove arrhythmic genesis foci and RF hyperthermia for cancer treatment [13]. Additionally, they are critical to accurately determine the specific absorption rate (tissue heating) induced by EM waves [1].

Several EP mapping approaches are explored to map the electrical properties of tissue in vivo. Electrical impedance tomography (EIT), for example, uses electrode mounting to detect currents injected into the sample [14]. This method is cost-effective and yields high temporal resolution, but poor spatial resolution due to the ill-posed nature of the inverse problem [13,15]. Magnetic induced tomography (MIT) applies an oscillating magnetic field to induce eddy currents in the object and detects the resulting magnetic fields outside the object [16]. However, it suffers from the same issues as EIT. Magnetic resonance electrical impedance tomography (MR-EIT) utilizes MRI to detect the magnetic field induced by the probing current [17]. This provides higher spatial resolution, but has a poor signal-to-noise ratio due to limitations on the amount of current injection [13,18,19]. Hall effect imaging (HEI) induces currents through surface electrodes and detects the emitted acoustic wave to reconstruct EPs [20]. This also has the potential to reach high resolution images, but all of the current injection based methods may suffer from shielding artifacts of non-conductivity tissue. Magneto-acoustic tomography with magnetic induction (MAT-MI) circumvents this shielding problem by inducing acoustic signals with time varying magnetic fields which are detected with ultrasound measurements [21]. However, methods that involve acoustic measurements are often limited to the surface of the object.

Electrical properties tomography (EPT) non-invasively images the conductivity and permittivity maps (simultaneously) in vivo from the radio frequency field signals obtained with MRI. The method does not require electrode mounting, does not induce additional external energy other than the inherent RF fields, and the RF fields can easily penetrate into most biological tissue. It uses a standard MRI system with regular RF coils. This concept was first introduced in 1991 by Haacke et al. [22] and first demonstrated in 2003 by Wen et al. [23]. The topic, however, only recently gained considerable interest by various research groups [1,13,19,24].

Several review papers discuss existing methods and review clinical applications [1,13,19,24]. These reviews, however, do not discuss the mathematical methodology in depth, which hampers the overview in terms of intrinsic assumptions. Here, however, a mathematical description of the acquired MR data and several differential and integral EPT approaches are described more thoroughly to give clear insights into the relations and differences between a large number of methods. This review thereby allows accurate comparisons between different methods and outlines their relative strengths and weaknesses. Extensions and generalizations are also mentioned. The EPT approaches are harmonized in terms of mathematical formulation, while maintaining as much as possible the structure of the original implementations to keep the transition from this manuscript to the references straightforward.

This review manuscript is organized as follows. First, general RF background information is presented together with a general formulation of an acquired MR image, which are necessary to understand some of the problems arising in EPT data acquisition. Second, some fundamental EPT equations are presented, from which the bulk of the analytical EPT approaches can be derived. The subsequent two sections discuss a large number of physical model-based EPT strategies, starting with methods that are based on transmit field data, followed by receive field-based methods. The review continues with a discussion about training-based EPT approaches. Finally, a general discussion is provided and EPT reconstruction examples are presented.

## 2. Phasor Representations for the RF Field

In EPT, knowledge about the RF field within the body is used to retrieve the dielectric properties (conductivity and permittivity) of tissue. This RF field is called the $B_{1}$ field and phasors are typically used to describe its behavior. What may lead to confusion is that there are actually two time conventions in common use to represent the RF field in terms of phasors. In particular, for a given time-domain RF field $B_{1}\left(r,t\right)$ operating at a frequency ω>0, phasors are introduced via the representation

(1) B1(r,t)=ReB^1(r,−iω)exp(−iωt)

or alternatively the representation

(2) B1(r,t)=ReB^1(r,iω)exp(iωt)

is used to describe the RF field. The vector ${\hat{B}}_{1}\left(r,-i\omega \right)$ is the phasor of the RF field when the time factor exp(−iωt) is used, while ${\hat{B}}_{1}\left(r,i\omega \right)$ is the phasor of the RF field in the situation where a time factor exp(iωt) is used.

For a given time convention, the phasor that corresponds to a given RF field is unique, and, since the time-domain RF field $B_{1}\left(r,t\right)$ is real-valued, the phasors of the two representations are related by

(3) $${\hat{B}}_{1}^{*}\left(r,-i\omega \right)={\hat{B}}_{1}\left(r,i\omega \right),$$

where the asterisk denotes complex conjugation. In other words, for a given RF field, the phasors of the two representations are the complex conjugate of each other. We note that if Equation (2) is used to represent RF fields, the letter j is often used for the imaginary unit instead of the letter i. We adopt this notation here as well and write the representation of Equation (2) as

(4) B1(r,t)=ReB^1(r,jω)exp(jωt).

Unless otherwise stated, we use the phasor representation of Equation (4) to describe the RF field.

Suppose now that we orient our reference frame such that the static background field $B_{0}$ is directed in the longitudinal *z*-direction ($B_{0}=\pm B_{0}\left(r\right)i_{z}$, $B_{0}\left(r\right)>0$) and we have available a transverse RF field with *x*- and *y*-components only. The corresponding phasor of this RF field is given by

(5) $${\hat{B}}_{1}\left(r,j\omega \right)={\hat{B}}_{1;x}\left(r,j\omega \right)i_{x}+{\hat{B}}_{1;y}\left(r,j\omega \right)i_{y},$$

which can be written as

(6) $${\hat{B}}_{1}\left(r,j\omega \right)={\hat{B}}_{1}^{+}\left(r,j\omega \right)+{\hat{B}}_{1}^{-}\left(r,j\omega \right)$$

with

(7) $${\hat{B}}_{1}^{+}\left(r,j\omega \right)={\hat{B}}_{1}^{+}\left(r,j\omega \right)\left(i_{x}-ji_{y}\right)\;\mathrm{and}\;{\hat{B}}_{1}^{-}\left(r,j\omega \right)={\left[{\hat{B}}_{1}^{-}\left(r,j\omega \right)\right]}^{*}\left(i_{x}+ji_{y}\right),$$

where we have introduced the ${\hat{B}}_{1}^{+}$ and ${\hat{B}}_{1}^{-}$ fields defined as

(8) $${\hat{B}}_{1}^{+}\left(r,j\omega \right)=\frac{{\hat{B}}_{1;x}\left(r,j\omega \right)+j{\hat{B}}_{1;y}\left(r,j\omega \right)}{2}$$

and

(9) $${\hat{B}}_{1}^{-}\left(r,j\omega \right)={\left[\frac{{\hat{B}}_{1;x}\left(r,j\omega \right)-j{\hat{B}}_{1;y}\left(r,j\omega \right)}{2}\right]}^{*},$$

respectively. Substitution of Equation (6) into Equation (4) leads to the time-domain RF field decomposition

(10) $$B_{1}\left(r,t\right)=B_{1}^{+}\left(r,t\right)+B_{1}^{-}\left(r,t\right)$$

with

(11) B1+(r,t)=ReB^1+(r,jω)exp(jωt)andB1−(r,t)=ReB^1−(r,jω)exp(jωt).

Finally, we decompose the scalar ${\hat{B}}_{1}^{+}$ and ${\hat{B}}_{1}^{-}$ fields into their real and imaginary parts as

B^1±=ReB^1±+jImB^1±.

Using these decompositions in Equation (7) and substituting the results in the field expressions for $B_{1}^{+}\left(r,t\right)$ and $B_{1}^{-}\left(r,t\right)$ as given by Equation (11) gives

(12) B1+(r,t)=ReB^1+cos(ωt)ix+sin(ωt)iy+ImB^1+−sin(ωt)ix+cos(ωt)iy

and

(13) B1−(r,t)=ReB^1−cos(ωt)ix−sin(ωt)iy+ImB^1−sin(ωt)ix+cos(ωt)iy.

From the above expressions, we observe that the $B_{1}^{+}$ vector traces out a circle in the transverse xy-plane and the radius of this circle is given by $|B_{1}^{+}\left|=\right|{\hat{B}}_{1}^{+}|$. The $B_{1}^{-}$ vector also traces out a circle but this circle has a radius $|B_{1}^{-}\left|=\right|{\hat{B}}_{1}^{-}|$ and is traversed in the opposite direction compared with the direction in which the circle of the $B_{1}^{+}$ field is traversed. Since in both cases the fields trace out a circle in the transverse plane, the $B_{1}^{+}$ and $B_{1}^{-}$ fields are called circularly polarized.

The direction of rotation depends upon the direction of the static background field. In particular, assume that our reference frame is such that the static $B_{0}$ field is in the negative *z*-direction: $B_{0}=-B_{0}\left(r\right)i_{z}$ with $B_{0}\left(r\right)>0$. From Equations (12) and (13), we observe that in this case the $B_{1}^{+}$ and $B_{1}^{-}$ fields rotate, respectively, in a left- and right-handed manner about the $B_{0}$ field. When the background field is directed in the positive $i_{z}$-direction, the situation is reversed and the circularly polarized fields $B_{1}^{+}$ and $B_{1}^{-}$ rotate, respectively, in a right- and left-handed manner about the $B_{0}$ field.

To summarize, any transverse RF field can be decomposed into two circularly polarized fields, where one is polarized in a left-handed manner with respect to background field, while the other is polarized in a right-handed manner with respect to the background field. Explicitly, we have

(14) $$B_{1}\left(r,t\right)=B_{1}^{\mathrm{lh}}\left(r,t\right)+B_{1}^{\mathrm{rh}}\left(r,t\right),$$

where $B_{1}^{\mathrm{lh}}\left(r,t\right)$ and $B_{1}^{\mathrm{rh}}\left(r,t\right)$ rotate in a left- and right-handed manner about the $B_{0}$ field, respectively. In the case that this background field is in the negative $i_{z}$-direction, we have

$$B_{1}^{\mathrm{lh}}\left(r,t\right)=B_{1}^{+}\left(r,t\right)\;\mathrm{and}\;B_{1}^{\mathrm{rh}}\left(r,t\right)=B_{1}^{-}\left(r,t\right),$$

while if the background field is in the positive $i_{z}$-direction we have

$$B_{1}^{\mathrm{lh}}\left(r,t\right)=B_{1}^{-}\left(r,t\right)\;\mathrm{and}\;B_{1}^{\mathrm{rh}}\left(r,t\right)=B_{1}^{+}\left(r,t\right).$$

### 2.1. Transmit and Receive Fields

As is well known, a circularly polarized RF field that operates at the Larmor frequency and that also rotates in a left-handed manner about the $B_{0}$ field influences the orientation of the magnetization, which ultimately leads to measurable MR signals. During transmission then, the left-handed circularly polarized part of the RF field, $B_{1}^{\mathrm{lh}}$, is of interest. In the case that the background field is in the negative $i_{z}$-direction, it is the scalar ${\hat{B}}_{1}^{+}$ field that determines $B_{1}^{\mathrm{lh}}$, while, if the background field is in the positive $i_{z}$-direction, it is the scalar field ${\hat{B}}_{1}^{-}$ that determines $B_{1}^{\mathrm{lh}}$.

Now, in the MRI literature, the left-handed circularly polarized RF field is always described in terms of a scalar ${\hat{B}}_{1}^{+}$ field, which seems to contradict the above observation that this field is described by ${\hat{B}}_{1}^{-}$ in the case that the static background field is in the positive $i_{z}$-direction. It is important, however, to realize that the scalar ${\hat{B}}_{1}^{+}$ and ${\hat{B}}_{1}^{-}$ fields are defined in terms of phasors that correspond to a particular time factor that is used to represent the RF field. Moreover, the phasors of the same RF field that correspond to the two different time factors are the complex-conjugate of each other (Equation (3)). Consequently, we have

$$\begin{array}{ccc} {\hat{B}}_{1}^{+}\left(r,j\omega \right) & =\frac{{\hat{B}}_{1;x}\left(r,j\omega \right)+j{\hat{B}}_{1;y}\left(r,j\omega \right)}{2} & \\ & =\frac{{\hat{B}}_{1;x}\left(r,i\omega \right)+i{\hat{B}}_{1;y}\left(r,i\omega \right)}{2}\; & (\mathrm{writing}\;i\;\mathrm{instead}\;\mathrm{of}\;j)\\ & =\frac{{\hat{B}}_{1;x}^{*}\left(r,-i\omega \right)+i{\hat{B}}_{1;y}^{*}\left(r,-i\omega \right)}{2}\; & (\mathrm{using}\;\mathrm{Equation}\;(3))\\ & ={\left[\frac{{\hat{B}}_{1;x}\left(r,-i\omega \right)-i{\hat{B}}_{1;y}\left(r,-i\omega \right)}{2}\right]}^{*}={\hat{B}}_{1}^{-}\left(r,-i\omega \right)\; \end{array}$$

and

$$\begin{array}{ccc} {\hat{B}}_{1}^{-}\left(r,j\omega \right) & ={\left[\frac{{\hat{B}}_{1;x}\left(r,j\omega \right)-j{\hat{B}}_{1;y}\left(r,j\omega \right)}{2}\right]}^{*} & \\ & =\frac{{\hat{B}}_{1;x}^{*}\left(r,j\omega \right)+j{\hat{B}}_{1;y}^{*}\left(r,j\omega \right)}{2}\; & \\ & =\frac{{\hat{B}}_{1;x}\left(r,-j\omega \right)+j{\hat{B}}_{1;y}^{*}\left(r,-j\omega \right)}{2}\; & (\mathrm{using}\;\mathrm{Equation}\;(3))\\ & =\frac{{\hat{B}}_{1;x}\left(r,-i\omega \right)+i{\hat{B}}_{1;y}\left(r,i\omega \right)}{2}={\hat{B}}_{1}^{+}\left(r,-i\omega \right)\; & (\mathrm{writing}\;i\;\mathrm{instead}\;\mathrm{of}\;j). \end{array}$$

In other words, the ${\hat{B}}_{1}^{+}$ field always describes a left-handed circularly polarized field provided that the phasors of Equation (4) are used for $B_{0}$ defined in the negative *z*-direction, while the phasors of Equation (1) have to be used for $B_{0}$ defined in the positive *z*-direction. Since transmitting a left-handed circularly polarized field operating at the Larmor frequency enables us to manipulate the magnetization, the ${\hat{B}}_{1}^{+}$ field is often referred to as the transmit field. Similarly, received signals can be expressed in terms of the right-handed circularly polarized field $B_{1}^{\mathrm{rh}}$, which is completely described by the ${\hat{B}}_{1}^{-}$ field if the phasors of Equation (4) are used in its definition for $B_{0}$ defined in the negative $i_{z}$-direction and the phasors of Equation (1) are used if the background field is in the positive $i_{z}$-direction. For this reason, the ${\hat{B}}_{1}^{-}$ field is often referred to as the receive field.

### 2.2. MR Imaging

**Figure 1:**
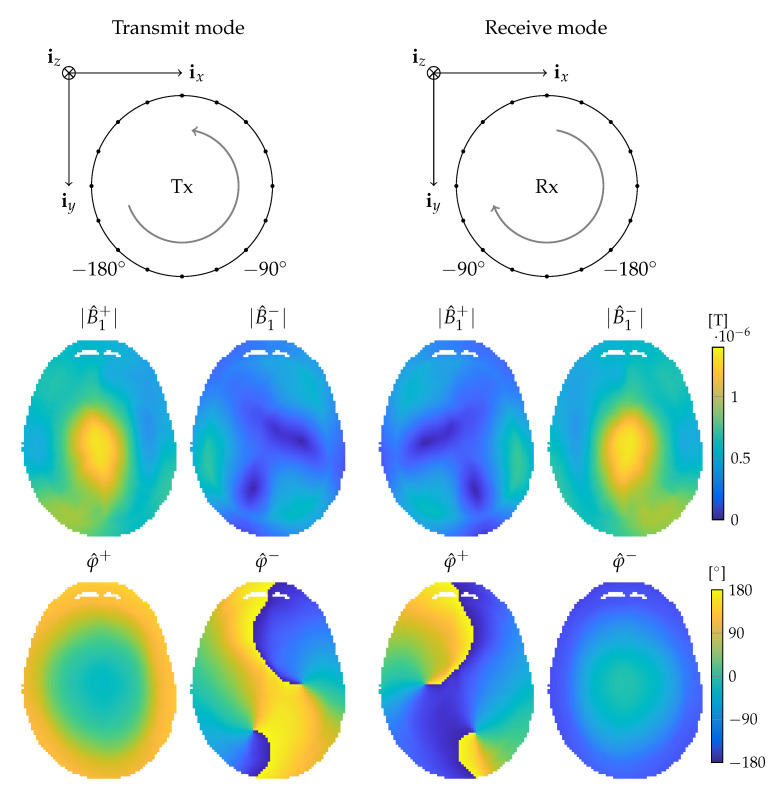
The transmit and receive mode of a tuned 16 rung 7 T MR head coil loaded with the Duke body model [29] and their corresponding transmit and receive fields (magnitude and phase).

The transmit field can be written in polar form as ${\hat{B}}_{1}^{+}=\left|{\hat{B}}_{1}^{+}\right|\exp\left(j{\hat{\varphi }}^{+}\right)$, where $\left|{\hat{B}}_{1}^{+}\right|$ is the amplitude or magnitude of the transmit field and ${\hat{\varphi }}^{+}\in \left(-\pi ,\pi \right]$ its phase. Similarly, the receive field can be written in polar form as ${\hat{B}}_{1}^{-}=\left|{\hat{B}}_{1}^{-}\right|\exp\left(j{\hat{\varphi }}^{-}\right)$, with $\left|{\hat{B}}_{1}^{-}\right|$ its amplitude and ${\hat{\varphi }}^{-}\in \left(-\pi ,\pi \right]$ its phase. Note that, to define a phase that is unique, we have restricted the transmit phase ${\hat{\varphi }}^{+}$ and the receive phase ${\hat{\varphi }}^{-}$ to the principle branch (−π,π]. Spatial information is encoded into the signal using magnetic field gradients, applied after the ${\hat{B}}_{1}^{+}$ field has tipped the magnetization into the transverse plane. Due to the interaction with the body, the transmit field has a spatial dependence, denoted ${\hat{B}}_{1}^{+}\left(r\right)$. The polar decomposition is used to express the acquired spatially dependent MR image as [13,19,24,25]

(15) $$I\left(r\right)=\varrho_{0}\left(r\right)\sin\left(\gamma \tau \left|{\hat{B}}_{1}^{+}\left(r\right)\right|\right)\exp\left[j{\hat{\varphi }}^{+}\left(r\right)\right]{\hat{B}}_{1}^{-;*}\left(r\right),$$

with $\varrho_{0}$ the proton density, γ the gyromagnetic ratio and τ the RF pulse duration and where ${\hat{B}}_{1}^{-;*}$ is the complex conjugate of ${\hat{B}}_{1}^{-}$. In this simplified expression for the acquired MR image, system dependent factors and contrast terms that underlie an MR image, such as $T_{1}$ and $T_{2}$ relaxation, are ignored. Of the transmit and receive fields, only the magnitude of the transmit field shows a non-linear impact on the MR image. This non-linear relation allows for the direct measurement of the transmit magnitude by combining images from different scans such that confounding factors cancel. However, the acquired phase is always the superposition of the phases of ${\hat{B}}_{1}^{+}$ and ${\hat{B}}_{1}^{-;*}$, called the transceive phase, which can not be disentangled from measurements and are therefore difficult to determine exactly. It has been observed that at 1.5 and 3 T the transmit phase closely resembles the phase of ${\hat{B}}_{1}^{-;*}$ (see also the example given in Figure 1), and in those cases the transmit phase is therefore typically estimated as half the transceive phase: this is termed the transceive phase assumption [23,26]. Similarly, the (magnitude of the) receive field is weighted by the proton density, which is also difficult to disentangle. If the proton-density is not negligible, the proton-density or magnitude of the receive field can be extracted from their product term based on symmetry patterns of the transmit and receive fields in the case of a symmetrical object and imaging setup [27,28]. Additionally, the proton-density could be removed via suitable modeling based on image segmentation [27]. However, knowledge of the transmit phase, receive phase or receive magnitude individually is not always necessary, but could also potentially be determined through EPT.

### 2.3. Transmit and Receive Fields in Terms of Measurable Quantities

Consider a multi-element RF antenna with *P* transmit and *Q* receive channels. The transmit field from channel *p* (${\hat{B}}_{1p}^{+}$) measured at receive channel *q* can then be written in measurable (known) and unknown terms as

(16) $$\begin{array}{cc} {\hat{B}}_{1p}^{+} & =\left|{\hat{B}}_{1p}^{+}\right|\exp\left[j\left({\hat{\varphi }}_{p}^{+}-{\hat{\varphi }}_{q}^{-}\right)\right]\exp\left(j{\hat{\varphi }}_{q}^{-}\right)\\ & =\left|{\hat{B}}_{1p}^{+}\right|\exp\left(j{\hat{\varphi }}_{pq}^{\pm }\right)\exp\left(j{\hat{\varphi }}_{q}^{-}\right)\\ & ={\hat{B}}_{1p}^{+;\mathrm{TRX}(p,q)}\exp\left(j{\hat{\varphi }}_{q}^{-}\right) \end{array}$$

with ${\hat{\varphi }}_{pq}^{\pm }={\hat{\varphi }}_{p}^{+}-{\hat{\varphi }}_{q}^{-}$ the transceive phase (note that ${\hat{\varphi }}^{-}$ is sometimes defined as the argument of ${\hat{B}}_{1}^{-;*}$, such that the transceive phase is given by ${\hat{\varphi }}^{\pm }={\hat{\varphi }}^{+}+{\hat{\varphi }}^{-}$), ${\hat{\varphi }}_{p}^{+}$ the absolute transmit phase of transmit channel *p* and ${\hat{\varphi }}_{q}^{-}$ the absolute receive phase of receive channel *q*. The term ${\hat{B}}_{1p}^{+;\mathrm{TRX}(p,q)}=\left|{\hat{B}}_{1p}^{+}\right|\exp\left(j{\hat{\varphi }}_{pq}^{\pm }\right)$ is the measurable term, while $\exp\left(j{\hat{\varphi }}_{q}^{-}\right)$ is the unknown term. Note that this formulation is applicable for RF coils in general with P=Q=1, while the subsequent two formulations in terms of relative transmit phases are only applicable for multi-element RF arrays with P>1 and/or Q>1. The transmit field from channel *p* can be written in terms of relative phase distributions as

(17) $$\begin{array}{cc} {\hat{B}}_{1p}^{+} & =\left|{\hat{B}}_{1p}^{+}\right|\exp\left[j\left({\hat{\varphi }}_{p}^{+}-{\hat{\varphi }}_{r}^{+}\right)\right]\exp\left(j{\hat{\varphi }}_{r}^{+}\right)\\ & =\left|{\hat{B}}_{1p}^{+}\right|\exp\left(j{\hat{\varphi }}_{pr}^{+}\right)\exp\left(j{\hat{\varphi }}_{r}^{+}\right)\\ & ={\hat{B}}_{1p}^{+;\mathrm{rel}(p,r)}\exp\left(j{\hat{\varphi }}_{r}^{+}\right) \end{array}$$

with ${\hat{\varphi }}_{pr}^{+}={\hat{\varphi }}_{p}^{+}-{\hat{\varphi }}_{r}^{+}$ the transmit phase of channel *p* relative to the reference transmit phase ${\hat{\varphi }}_{r}^{+}$ of channel *r*. ${\hat{B}}_{1p}^{+;\mathrm{rel}(p,r)}=\left|{\hat{B}}_{1p}^{+}\right|\exp\left(j{\hat{\varphi }}_{pr}^{+}\right)$ is the measurable term, while $\exp\left(j{\hat{\varphi }}_{r}^{+}\right)$ is the unknown term. Additionally, the receive phase can also be written in a similar formulation. The measurable term is, however, weighted by the proton density. For the conjugate of the receive field, we have

(18) $$\begin{array}{cc} {\hat{B}}_{1q}^{-;*} & =\left|\varrho_{0}{\hat{B}}_{1q}^{-}\right|\exp\left[j\left(-{\hat{\varphi }}_{q}^{-}+{\hat{\varphi }}_{r}^{-}\right)\right]\left|\varrho_{0}^{-1}\right|\exp\left(-j{\hat{\varphi }}_{r}^{-}\right)\\ & =\left|\varrho_{0}{\hat{B}}_{1q}^{-}\right|\exp\left(j{\hat{\varphi }}_{rq}^{-}\right)\left|\varrho_{0}^{-1}\right|\exp\left(-j{\hat{\varphi }}_{r}^{-}\right) \end{array}$$

with ${\hat{\varphi }}_{rq}^{-}={\hat{\varphi }}_{r}^{-}-{\hat{\varphi }}_{q}^{-}$ the receive phase of reference channel *r* relative to channel *q*. $\left|\varrho_{0}{\hat{B}}_{1q}^{-}\right|\exp\left(j{\hat{\varphi }}_{rq}^{-}\right)$ is the measurable term, while $\left|\varrho_{0}^{-1}\right|\exp\left(-j{\hat{\varphi }}_{r}^{-}\right)$ is the unknown term.

In summary, we use the phasor representation of Equation (4) to describe the RF field and orient the $B_{0}$ field in the negative $i_{z}$-direction such that ${\hat{B}}_{1}^{+}$ enables the manipulation of magnetization. Furthermore, we describe the transmit and receive fields with Equations (8) and (9), such that ${\hat{B}}_{1}^{+}$ and ${\hat{B}}_{1}^{-}$ described by the phasor representation of Equation (4) correspond to the ${\hat{B}}_{1}^{-}$ and ${\hat{B}}_{1}^{+}$ fields described by the phasor representation of Equation (1), respectively. Additionally, the MR image can be described by Equation (15), which shows that the transmit and receive field phases, as well as the proton density and the receive field magnitude, are entangled and therefore not directly available from MR acquisitions. Instead of making assumptions about the acquirable data to obtain absolute transmit or receive field maps, the transmit and receive fields can also be expressed in terms of known (directly derived from measurements) and unknown terms, as depicted in Equations (16)–(18).

## 3. Fundamental EPT Equations

Physical model-based EPT approaches all rely on a few fundamental equations from which their central equations are derived. To derive and understand the approaches, knowledge about the Maxwell’s equations, the Helmholtz equation and the scattering field formalism is required. These are summarized below.

### 3.1. First-Order Differential Equations: Maxwell’s Equations

Maxwell’s equations for time-harmonic fields are given by

(19) $$\begin{array}{cc} -\nabla \times \hat{H}\left(r\right)+\eta \left(r\right)\hat{E}\left(r\right) & =-{\hat{J}}^{\mathrm{ext}}\left(r\right),\;\mathrm{and} \end{array}$$

(20) $$\begin{array}{cc} \nabla \times \hat{E}\left(r\right)+\zeta \left(r\right)\hat{H}\left(r\right) & =0, \end{array}$$

with $\eta \left(r\right)=\sigma \left(r\right)+j\omega \varepsilon \left(r\right)$ and $\zeta \left(r\right)=j\omega \mu \left(r\right)$, which are, respectively, the per-unit-length admittance and impedance of the medium. Here, σ, ε, μ and ω are the conductivity, permittivity, permeability and angular (RF) frequency, respectively. Additionally, in the MR setting, ${\hat{J}}^{\mathrm{ext}}$ is an external current density distribution present on the MR coil that generates the EM fields. Since these sources are located outside the body and since the permeability of biological tissue is assumed to be constant and equal to that of vacuum, the RF field inside the body satisfies the Maxwell equations

(21) $$\begin{array}{cc} -\nabla \times \hat{B}\left(r\right)+\mu_{0}\eta \left(r\right)\hat{E}\left(r\right) & =0,\;\mathrm{and} \end{array}$$

(22) $$\begin{array}{cc} \nabla \times \hat{E}\left(r\right)+j\omega \hat{B}\left(r\right) & =0, \end{array}$$

with $\hat{B}=\mu_{0}\hat{H}$. Furthermore, introducing the vectors

$$i^{+}=\frac{1}{2}\left(i_{x}+ji_{y}\right)\;\mathrm{and}\;i^{-}=\frac{1}{2}\left(i_{x}-ji_{y}\right)$$

we have for the transmit and receive fields the expressions ${\hat{B}}_{1}^{+}=i^{+}\cdot \hat{B}$ and ${\hat{B}}_{1}^{-;*}=i^{-}\cdot \hat{B}$. Similarly, we define ${\hat{E}}_{1}^{+}=i^{+}\cdot \hat{E}$ and ${\hat{E}}_{1}^{-;*}=i^{-}\cdot \hat{E}$ and introduce the differentiation operators (Wirtinger derivatives)

(23) $$\partial^{+}=i^{+}\cdot \nabla =\frac{1}{2}\left(\partial_{x}+j\partial_{y}\right)\;\mathrm{and}\;\partial^{-}=i^{-}\cdot \nabla =\frac{1}{2}\left(\partial_{x}-j\partial_{y}\right).$$

Taking the inner product of $i^{+}$ and the second Maxwell equation now gives an explicit expression for the transmit field, while taking the inner product of $i^{-}$, and this second Maxwell equation gives an explicit expression for the receive field. Explicitly, we have

(24) $${\hat{B}}_{1}^{+}=\frac{1}{\omega }\left(\partial^{+}E_{z}-\partial_{z}{\hat{E}}_{1}^{+}\right)\;\mathrm{and}\;{\hat{B}}_{1}^{-;*}=-\frac{1}{\omega }\left(\partial^{-}E_{z}-\partial_{z}{\hat{E}}_{1}^{-;*}\right).$$

These relations tell us that the ${\hat{B}}_{1}^{+}$ and ${\hat{B}}_{1}^{-;*}$ fields result from a difference between transverse variations of the longitudinal electric field (as determined by the Wirtinger derivatives) and longitudinal variations of the transverse ${\hat{E}}_{1}^{+}$ and ${\hat{E}}_{1}^{-;*}$ fields. These equations are used as a starting point in the EPT method discussed in Section 4.7. For completeness, we mention that, if a similar procedure is followed for the first Maxwell equation, we obtain

(25) $${\hat{E}}_{1}^{+}=\frac{1}{j\mu_{0}\eta }\left(\partial^{+}B_{z}-\partial_{z}{\hat{B}}_{1}^{+}\right)\;\mathrm{and}\;{\hat{E}}_{1}^{-;*}=-\frac{1}{j\mu_{0}\eta }\left(\partial^{-}B_{z}-\partial_{z}{\hat{B}}_{1}^{-;*}\right).$$

### 3.2. Second-Order Differential Equation: The Generalized Helmholtz Equation

Since the objective is to obtain the dielectric tissue parameters from magnetic field data, a second option is not to consider the electric field at all and to eliminate this field from the source-free first-order Maxwell system as given by Equations (21) and (22). To this end, we take the curl of Equation (21) and obtain

(26) $$-\nabla \times \nabla \times \hat{B}+\mu_{0}\nabla \times \left(\eta \hat{E}\right)=0.$$

Since

$$\begin{array}{c} \nabla \times \nabla \times \hat{B}=\nabla \nabla \cdot \hat{B}-\nabla^{2}\hat{B} \end{array}$$

and

$$\begin{array}{c} \nabla \times \left(\eta \hat{E}\right)=\nabla \eta \times \hat{E}+\eta \nabla \times \hat{E}=\frac{1}{\mu_{0}}\frac{\nabla \eta }{\eta }\times \left(\nabla \times \hat{B}\right)-j\omega \eta \hat{B}, \end{array}$$

Equation (26) can be written as

$$-\nabla \nabla \cdot \hat{B}+\nabla^{2}\hat{B}+\frac{\nabla \eta }{\eta }\times \left(\nabla \times \hat{B}\right)-\eta \zeta \hat{B}=0.$$

Finally, taking the divergence of the second Maxwell equation (Equation (22)), we get $\nabla \cdot \hat{B}=0$ and substituting this result in the above equation, we obtain the generalized Helmholtz equation

(27) $$\nabla^{2}\hat{B}+\frac{\nabla \eta }{\eta }\times \left(\nabla \times \hat{B}\right)+k^{2}\hat{B}=0,$$

where $k={\left(-\eta \zeta \right)}^{1/2}={\left(\omega^{2}\mu_{0}\varepsilon -j\omega \mu_{0}\sigma \right)}^{1/2}$ is the complex wave number with Im(k)≤0. Note that, for homogeneous media, η is constant and the second term on the left-hand side vanishes. In this case, we have the Helmholtz equation

(28) $$\nabla^{2}\hat{B}+k^{2}\hat{B}=0.$$

Taking the inner product of the vector $i^{+}$ and Equation (28) gives the Helmholtz equation for the ${\hat{B}}_{1}^{+}$ field

(29) $$\nabla^{2}{\hat{B}}_{1}^{+}+k^{2}{\hat{B}}_{1}^{+}=0,$$

which serves as a starting point for the EPT methods discussed in Section 4.1, Section 4.2, Section 4.3, Section 5.1 and Section 5.2. It is important to realize that the above Helmholtz equation is valid for homogeneous media (η is constant) only. For general inhomogeneous media (η is not constant), we have the generalized Helmholtz equation (Equation (27)). Dotting this equation with the vector $i^{+}$, we end up with the generalized Helmholtz equation for the ${\hat{B}}_{1}^{+}$ field given by

(30) $$\nabla^{2}{\hat{B}}_{1}^{+}+i^{+}\cdot \left[\frac{\nabla \eta }{\eta }\times \left(\nabla \times \hat{B}\right)\right]+k^{2}{\hat{B}}_{1}^{+}=0.$$

This equation serves as a starting point for the EPT methods discussed in Section 4.4, Section 4.5 and Section 4.6, but with the second term on the left-hand side rewritten in terms of ${\hat{B}}_{1}^{+}$ and ${\hat{B}}_{z}$. Specifically, in the EPT methods of Section 4.4 and Section 4.5, the generalized Helmholtz equation is rewritten in terms of the gradient of ${\hat{B}}_{1}^{+}$ and ${\hat{B}}_{z}$, while, in the methods of Section 4.6, the generalized Helmholtz equation is written as a convection–reaction equation.

#### 3.2.1. The Gradient-Type Generalized Helmholtz Equation

Let us first consider rewriting the generalized Helmholtz equation (Equation (30)) in terms of the gradient of the ${\hat{B}}_{1}^{+}$ field and ${\hat{B}}_{z}$, the *z*-component of the magnetic field. As a first step, we introduce the vector $g=\eta^{-1}\nabla \eta$ and write $g^{+}=i^{+}\cdot g$. The second term on the left-hand side in Equation (30) can now be written as

$$\begin{array}{cc} i^{+}\cdot \left[\frac{\nabla \eta }{\eta }\times \left(\nabla \times \hat{B}\right)\right]\; & =i^{+}\cdot \left[g\times \left(\nabla \times \hat{B}\right)\right]=g\cdot \left(\partial^{+}\hat{B}-\nabla {\hat{B}}_{1}^{+}\right)\\ \; & =g_{x}\left(\partial^{+}{\hat{B}}_{x}-\partial_{x}{\hat{B}}_{1}^{+}\right)+g_{y}\left(\partial^{+}{\hat{B}}_{y}-\partial_{y}{\hat{B}}_{1}^{+}\right)+g_{z}\left(\partial^{+}{\hat{B}}_{z}-\partial_{z}{\hat{B}}_{1}^{+}\right). \end{array}$$

Since

$$\partial^{+}{\hat{B}}_{x}-\partial_{x}{\hat{B}}_{1}^{+}=-j\left(\partial^{+}{\hat{B}}_{y}-\partial_{y}{\hat{B}}_{1}^{+}\right)=\frac{1}{2}j\left(\partial_{y}{\hat{B}}_{x}-\partial_{x}{\hat{B}}_{y}\right),$$

this can be written as

(31) $$i^{+}\cdot \left[\frac{\nabla \eta }{\eta }\times \left(\nabla \times \hat{B}\right)\right]=\left(jg_{x}-g_{y}\right)\;\frac{1}{2}\left(\partial_{y}{\hat{B}}_{x}-\partial_{x}{\hat{B}}_{y}\right)+g_{z}\left(\partial^{+}{\hat{B}}_{z}-\partial_{z}{\hat{B}}_{1}^{+}\right).$$

Furthermore, using $\nabla \cdot \hat{B}=0$, we have

(32) $$\frac{1}{2}\left(\partial_{y}{\hat{B}}_{x}-\partial_{x}{\hat{B}}_{y}\right)=j\partial_{x}{\hat{B}}_{1}^{+}+\partial_{y}{\hat{B}}_{1}^{+}+\frac{1}{2}j\partial_{z}{\hat{B}}_{z}$$

and substituting this relation in Equation (31) leads to

$$i^{+}\cdot \left[\frac{\nabla \eta }{\eta }\times \left(\nabla \times \hat{B}\right)\right]=-f^{+}\cdot \nabla {\hat{B}}_{1}^{+}-h^{+}\cdot \nabla {\hat{B}}_{z}$$

with

$$f^{+}=4g^{+}i^{-}+g_{z}i_{z}\;\mathrm{and}\;h^{+}=-g_{z}i^{+}+g^{+}i_{z}.$$

With this result, we end up with the gradient-type generalized Helmholtz equation

(33) $$\nabla^{2}{\hat{B}}_{1}^{+}-f^{+}\cdot \nabla {\hat{B}}_{1}^{+}-h^{+}\cdot \nabla {\hat{B}}_{z}+k^{2}{\hat{B}}_{1}^{+}=0.$$

#### 3.2.2. The Generalized Helmholtz Equation as a Convection–Reaction Equation

To arrive at the convection–reaction form of the generalized Helmholtz equation as used in EPT, we return to Equation (32) and rewrite this equation as

(34) $$\frac{1}{2}\left(\partial_{y}{\hat{B}}_{x}-\partial_{x}{\hat{B}}_{y}\right)=j\partial_{x}{\hat{B}}_{1}^{+}+\partial_{y}{\hat{B}}_{1}^{+}+\frac{1}{2}j\partial_{z}{\hat{B}}_{z}=j\left(2\partial^{-}{\hat{B}}_{1}^{+}+\frac{1}{2}\partial_{z}{\hat{B}}_{z}\right).$$

Substitution of this result in Equation (31) gives

$$i^{+}\cdot \left[\frac{\nabla \eta }{\eta }\times \left(\nabla \times \hat{B}\right)\right]=-\beta^{+}\cdot g,$$

where

(35) $$\beta^{+}=\left(2\partial^{-}{\hat{B}}_{1}^{+}+\frac{1}{2}\partial_{z}{\hat{B}}_{z}\right)i_{x}+j\left(2\partial^{-}{\hat{B}}_{1}^{+}+\frac{1}{2}\partial_{z}{\hat{B}}_{z}\right)i_{y}+\left(\partial_{z}{\hat{B}}_{1}^{+}-\partial^{+}{\hat{B}}_{z}\right)i_{z}.$$

The generalized Helmholtz equation now becomes

$$\nabla^{2}{\hat{B}}_{1}^{+}-\beta^{+}\cdot g+k^{2}{\hat{B}}_{1}^{+}=0.$$

Dividing this equation by η and using the definition of vector g, we arrive at our final form

(36) $$u\nabla^{2}{\hat{B}}_{1}^{+}+\beta^{+}\cdot \nabla u-\zeta {\hat{B}}_{1}^{+}=0$$

with $u=\eta^{-1}$. Equation (36) is the generalized Helmholtz equation in convection–reaction form, where $u\nabla^{2}{\hat{B}}_{1}^{+}-\zeta {\hat{B}}_{1}^{+}$ is the reaction component and $\beta^{+}$ is the convective field. Observe that the components of the convective field are directly related to the dielectric medium parameters and the *z*-component of the electric field strength via (cf. Equations (24) and (25))

(37) $$2\beta_{x}=j\mu_{0}\eta {\hat{E}}_{z},\;2\beta_{y}=j2\beta_{x}=-\mu_{0}\eta {\hat{E}}_{z},\;\mathrm{and}\;\beta_{z}=-j\mu_{0}\eta {\hat{E}}^{+}.$$

These relations are used as a starting point in the EPT methods discussed in Section 4.7 and Section 4.8.

#### 3.2.3. Helmholtz Equations for the Receive Field

For completeness, we mention that a similar procedure can be carried out for the ${\hat{B}}_{1}^{-}$ field. In particular, taking the inner product of the vector $i^{-}$ and Equation (27), we end up with

(38) $$\nabla^{2}{\hat{B}}_{1}^{-;*}+i^{-}\cdot \left[\frac{\nabla \eta }{\eta }\times \left(\nabla \times \hat{B}\right)\right]+k^{2}{\hat{B}}_{1}^{-;*}=0,$$

which is the generalized Helmholtz equation for ${\hat{B}}_{1}^{-;*}$. This equation can also be written in terms of gradients of the ${\hat{B}}_{1}^{-;*}$ field and ${\hat{B}}_{z}$ as

(39) $$\nabla^{2}{\hat{B}}_{1}^{-;*}-f^{-}\cdot \nabla {\hat{B}}_{1}^{-;*}-h^{-}\cdot \nabla {\hat{B}}_{z}+k^{2}{\hat{B}}_{1}^{-;*}=0,$$

with

$$f^{-}=4g^{-}i^{+}+g_{z}i_{z}\;\mathrm{and}\;h^{-}=-g_{z}i^{-}+g^{-}i_{z},$$

where we introduce $g^{-}=i^{-}\cdot g$, or as a convection–reaction equation as

(40) $$u\nabla^{2}{\hat{B}}_{1}^{-;*}+\beta^{-}\cdot \nabla u-\zeta {\hat{B}}_{1}^{-;*}=0,$$

with

$$\beta^{-}=\left(2\partial^{+}{\hat{B}}_{1}^{-;*}+\frac{1}{2}\partial_{z}{\hat{B}}_{z}\right)i_{x}-j\left(2\partial^{+}{\hat{B}}_{1}^{-;*}+\frac{1}{2}\partial_{z}{\hat{B}}_{z}\right)i_{y}+\left(\partial_{z}{\hat{B}}_{1}^{-;*}-\partial^{-}{\hat{B}}_{z}\right)i_{z}.$$

For the vectorial Helmholtz equation of Equation (28), dotting with the vector $i^{-}$ gives

(41) $$\nabla^{2}{\hat{B}}_{1}^{-;*}+k^{2}{\hat{B}}_{1}^{-;*}=0,$$

which is the Helmholtz equation for the ${\hat{B}}_{1}^{-;*}$ field in the case of homogeneous media.

### 3.3. Volume Integral Equations

The fundamental integral equations are obtained through a scattering formalism by exploiting the linearity of Maxwell’s equations. Specifically, the total electromagnetic field in the presence of an object in an MR coil is denoted by $\left\{\hat{E},\hat{H}\right\}$, and this field is written as the sum of an incident and scattered field as

(42) $$\left\{\hat{E},\hat{H}\right\}=\left\{{\hat{E}}^{\mathrm{inc}},{\hat{H}}^{\mathrm{inc}}\right\}+\left\{{\hat{E}}^{\mathrm{sca}},{\hat{H}}^{\mathrm{sca}}\right\},$$

where the incident field is defined as the field that is present in an empty (air-filled) RF coil. This incident field is generated by an external current density distribution ${\hat{J}}^{\mathrm{ext}}$ representing the MR coil that occupies the bounded source domain S. The governing equations for the incident field are

(43) $${\hat{E}}^{\mathrm{inc}}=\left(k_{0}^{2}+\nabla \nabla \cdot \right){\hat{A}}^{\mathrm{ext}}\;\mathrm{and}\;{\hat{H}}^{\mathrm{inc}}=\eta_{0}\nabla \times {\hat{A}}^{\mathrm{ext}},$$

with $k_{0}=\omega /c_{0}$ the wave number of the background medium, $c_{0}$ the electromagnetic wave speed of free space and $\eta_{0}=j\omega \varepsilon_{0}$ the admittance of the background medium. In the above field expressions, the vector potential ${\hat{A}}^{\mathrm{ext}}$ is given by

$${\hat{A}}^{\mathrm{ext}}\left(r\right)=\eta_{0}^{-1}\int_{r^{\prime }\in S}\hat{G}\left(r-r^{\prime }\right){\hat{J}}^{\mathrm{ext}}\left(r^{\prime }\right)\;dV,$$

where $\hat{G}\left(r\right)$ is the Green’s function of the background medium given by

$$\hat{G}\left(r\right)=\frac{\exp\left(-jk_{0}\left|r\right|\right)}{4\pi \left|r\right|},\;\mathrm{for}\;r\neq 0.$$

When there is an object present, scattered fields will be generated due to an induced scattering current density distribution ${\hat{J}}^{\mathrm{sca}}$ having the object domain D as its support. The scattered fields are given by

(44) $${\hat{E}}^{\mathrm{sca}}=\left(k_{0}^{2}+\nabla \nabla \cdot \right){\hat{A}}^{\mathrm{sca}}\;\mathrm{and}\;{\hat{H}}^{\mathrm{sca}}=\eta_{0}\nabla \times {\hat{A}}^{\mathrm{sca}},$$

where the vector potential ${\hat{A}}^{\mathrm{sca}}$ is given by

$${\hat{A}}^{\mathrm{sca}}\left(r\right)=\eta_{0}^{-1}\int_{r^{\prime }\in D}\hat{G}\left(r-r^{\prime }\right){\hat{J}}^{\mathrm{sca}}\left(r^{\prime }\right)\;dV$$

with ${\hat{J}}^{\mathrm{sca}}=\left(\eta -\eta_{0}\right)\hat{E}$ the scattered current density distribution. Note that the scattering current density and consequently the scattered field vanish if the object is absent ($\eta =\eta_{0}$) and the total electromagnetic field is equal to the incident field. Finally, we mention that the ${\hat{B}}_{1}^{+}$ field can be obtained from the vector potential as

(45) $${\hat{B}}_{1}^{+}={\hat{B}}_{1}^{+;\mathrm{inc}}+{\hat{B}}_{1}^{+;\mathrm{sca}}\;\mathrm{with}\;{\hat{B}}_{1}^{+;\mathrm{inc}}=\frac{\omega }{c_{0}^{2}}\tilde{\nabla }\cdot {\hat{A}}^{\mathrm{ext}}\;\mathrm{and}\;{\hat{B}}_{1}^{+;\mathrm{sca}}=\frac{\omega }{c_{0}^{2}}\tilde{\nabla }\cdot {\hat{A}}^{\mathrm{sca}},$$

where $\tilde{\nabla }=i_{z}\partial^{+}-i^{+}\partial_{z}$. The dielectric tissue parameters only influence ${\hat{B}}_{1}^{+;\mathrm{sca}}$, that is, the effects of the medium parameters on the ${\hat{B}}_{1}^{+}$ field have been separated from the excited incident ${\hat{B}}_{1}^{+}$ field. These relations are used as starting point for the EPT methods discussed in Section 4.9, Section 4.10 and Section 4.11.

## 4. EPT Methods Requiring Transmit Field Mapping

This section discusses analytical EPT approaches based on transmit field mappings. The section starts with direct local differential methods and roughly transitions to end with forward global integral methods. More specifically, the EPT methods discussed in this section are

- Section 4.1: Helmholtz-based EPT (H-EPT)
- Section 4.2: Simplified H-EPT (SH-EPT)
- Section 4.2.1: Poisson-based Conductivity Mapping (P-CM)
- Section 4.3: Local Maxwell tomography (LMT)
- Section 4.4: Modified dual-excitation EPT (MDE-EPT)
- Section 4.5: Gradient-based EPT (G-EPT)
- Section 4.6: Convection–reaction EPT (CR-EPT)
- Section 4.6.1: Phase-only convection–reaction conductivity mapping (PCR-CM)
- Section 4.7: Transverse EPT (T-EPT)
- Section 4.8: First-order induced-current EPT (foIC-EPT)
- Section 4.9: Variational Born iterative method EPT (VBIM-EPT)
- Section 4.10: Global Maxwell tomography (GMT)
- Section 4.11: Contrast source inversion EPT (CSI-EPT)

Other methods that do not require transmit field mapping are discussed in Section 5. Machine-learning approaches are discussed in Section 6.

### 4.1. Helmholtz-Based EPT

Helmholtz-based EPT (H-EPT) assumes a homogeneous medium (∇η=0) and is based on the Helmholtz equation (Equation (29)) [22,23,30]. Explicitly, assuming that the ${\hat{B}}_{1}^{+}$ field is known, the tissue parameters are determined from

(46) $$\frac{\nabla^{2}{\hat{B}}_{1}^{+}}{{\hat{B}}_{1}^{+}}=-k^{2}$$

and the definition of the wave number as

(47) σ=1ωμ0Im∇2B^1+B^1+andε=−1ω2μ0Re∇2B^1+B^1+.

This explicit method is extremely simple, easy to implement and fast to compute. However, the homogeneity assumption results in errors at tissue boundaries; the second-order derivative that acts on the data makes the method sensitive to noise [31,32,33]; and the method requires knowledge of the absolute transmit phase which is not directly available. To mitigate noise effects, filtered Laplacians with increased kernel size can be used, however, this leads to a severe numerical boundary error propagation [32,34]. The second-order differential has been reduced to first-order derivatives in an alternative formulation based on Gauss’ integral theorem, but image segmentation is required to implement this method [35,36]. Since the absolute transmit phase is in practice unavailable, it is typically estimated with the transceive phase assumption. However, since the Laplacian of a variable is the divergence of the gradient of the variable, this assumption can be prevented with multiple acquisitions from a multi-element array. This system namely allows for the determination of the gradient of the transmit phase of a reference transmit channel ($\nabla {\hat{\varphi }}_{r}^{+}$) from relative transmit phases [37] or the gradient of the receive phase of the receive channel ($\nabla {\hat{\varphi }}_{q}^{-}$) from transceive phase measurements [27].

### 4.2. Simplified H-EPT

Simplified H-EPT (SH-EPT) derives the conductivity and permittivity independently from the phase and magnitude of the ${\hat{B}}_{1}^{+}$, respectively [8,35]. Starting point is again the Helmholtz equation (Equation (29)) for the ${\hat{B}}_{1}^{+}$ field. However, here the polar decomposition of ${\hat{B}}_{1}^{+}$ is substituted, which gives

(48) $$\frac{\nabla^{2}\left|{\hat{B}}_{1}^{+}\right|}{\left|{\hat{B}}_{1}^{+}\right|}-{\left|\nabla {\hat{\varphi }}^{+}\right|}^{2}+j\left(2\frac{\nabla \left|{\hat{B}}_{1}^{+}\right|}{\left|{\hat{B}}_{1}^{+}\right|}\cdot \nabla {\hat{\varphi }}^{+}+\nabla^{2}{\hat{\varphi }}^{+}\right)=-k^{2}$$

and, equating the real and imaginary parts in the above equation, we obtain

(49) $$\sigma =\frac{1}{\omega \mu_{0}}\left(2\frac{\nabla |{\hat{B}}_{1}^{+}|\cdot \nabla {\hat{\varphi }}^{+}}{\left|{\hat{B}}_{1}^{+}\right|}+\nabla^{2}{\hat{\varphi }}^{+}\right)\;\mathrm{and}\;\varepsilon =\frac{-1}{\omega^{2}\mu_{0}}\left(\frac{\nabla^{2}\left|{\hat{B}}_{1}^{+}\right|}{\left|{\hat{B}}_{1}^{+}\right|}-{\left|\nabla {\hat{\varphi }}^{+}\right|}^{2}\right).$$

Finally, assuming that $\nabla^{2}{\hat{\varphi }}^{+}>>2\frac{\nabla \left|{\hat{B}}_{1}^{+}\right|\cdot \nabla {\hat{\varphi }}^{+}}{\left|{\hat{B}}_{1}^{+}\right|}$, we obtain

(50) $$\sigma =\frac{1}{\omega \mu_{0}}\nabla^{2}{\hat{\varphi }}^{+}.$$

Note that, if the Helmholtz equation accurately describes the behavior of the ${\hat{B}}_{1}^{+}$ field and if the above approximation holds, then only the phase of the ${\hat{B}}_{1}^{+}$ field is required to determine the conductivity. We remark that, if we write the ${\hat{B}}_{1}^{-}$ field in polar form as well and follow similar steps as for the ${\hat{B}}_{1}^{+}$ field, we obtain from the Helmholtz Equation (41)

$$\sigma =-\frac{1}{\omega \mu_{0}}\nabla^{2}{\hat{\varphi }}^{-},$$

where ${\hat{\varphi }}^{-}$ is the phase of the ${\hat{B}}_{1}^{-}$ field. Consequently, if the transceive phase ${\hat{\varphi }}^{\pm }={\hat{\varphi }}^{+}-{\hat{\varphi }}^{-}$ is available, we have

$$\sigma =\frac{1}{2\omega \mu_{0}}\nabla^{2}{\hat{\varphi }}^{\pm }.$$

Similarly, the assumption $\frac{\nabla^{2}\left|{\hat{B}}_{1}^{+}\right|}{\left|{\hat{B}}_{1}^{+}\right|}>>{\left|\nabla {\hat{\varphi }}^{+}\right|}^{2}$ results in

(51) $$\varepsilon =\frac{-1}{\omega^{2}\mu_{0}}\frac{\nabla^{2}\left|{\hat{B}}_{1}^{+}\right|}{\left|{\hat{B}}_{1}^{+}\right|}.$$

Clearly, in this case only the magnitude of the ${\hat{B}}_{1}^{+}$ field is required to determine the permittivity.

This EPT method is similar to H-EPT, but allows for conductivity or permittivity mapping without requiring the availability of both the magnitude and phase of the ${\hat{B}}_{1}^{+}$ field if the corresponding additional assumptions hold. The validity of these assumptions need to be investigated further. If only one of the EP maps is required, this approach enables, for example, shorter acquisition times or an increase in signal-to-noise ratio (SNR) of the transmit field map.

#### 4.2.1. Poisson-Based Conductivity Mapping

Poisson-based conductivity mapping (P-CM) considers Equation (50) as a Poisson equation for the phase. More precisely, in P-CM, we consider the Poisson equation

$$\nabla^{2}{\hat{\phi }}^{+}=\omega \mu_{0}\sigma$$

on $R^{3}$ and observe that the right-handed side has the object domain D as its support. Requiring that $\hat{\varphi }$ decays sufficiently fast at infinity ($|\hat{\varphi }|$ decreases as 1/|r| uniformly in r/|r| as |r|→∞), we have

(52) $${\hat{\phi }}^{+}\left(r\right)=-\omega \mu_{0}\int_{r^{\prime }\in D}{\hat{G}}_{P}\left(r-r^{\prime }\right)\sigma \left(r^{\prime }\right)\;dV$$

where ${\hat{G}}_{P}\left(r\right)$ is the 3D static Green’s function given by

$${\hat{G}}_{P}\left(r\right)=\frac{1}{4\pi |r|},\;\mathrm{for}\;r\neq 0.$$

In P-CM, we assume that the phase of the transmit field ${\hat{\varphi }}^{+}$ is known within the object, let r∈D in Equation (52), set ${\hat{\phi }}^{+}\left(r\right)={\hat{\varphi }}^{+}\left(r\right)$ for r∈D and retrieve a conductivity profile by minimizing Equation (52) in a least-squares sense.

P-CM is an integral formulation of the methods described in [38,39]. Its global integral approach has an inherent noise suppression effect which makes this method more robust to noise than local differentiation methods. Additionally, the minimization process allows for the inclusion of regularization as well. However, the method has an increased computational complexity compared to differential Helmholtz-based EPT approaches.

### 4.3. Local Maxwell Tomography

The simplified form of Local Maxwell Tomography (LMT) assumes the availability of a multi-element array and substitutes the polar decomposition of the ${\hat{B}}_{1}^{+}$ field as presented in Equation (16) into the Helmholtz equation (Equation (29)) [40]. We then obtain

(53) $$\frac{\nabla^{2}{\hat{B}}_{1p}^{+;\mathrm{TRX}(p,q)}}{{\hat{B}}_{1p}^{+;\mathrm{TRX}(p,q)}}=2\left(\nabla {\hat{\varphi }}_{pq}^{\pm }-j\frac{\nabla \left|{\hat{B}}_{1p}^{+}\right|}{\left|{\hat{B}}_{1p}^{+}\right|}\right)\cdot \nabla {\hat{\varphi }}_{q}^{-}+\left({\left|\nabla {\hat{\varphi }}_{q}^{-}\right|}^{2}-j\nabla^{2}{\hat{\varphi }}_{q}^{-}\right)-k^{2}.$$

This local equation is assumed to hold inside the object domain D and can be written as

(54) $$a^{T}\left(r\right)x\left(r\right)=b\left(r\right)\;\mathrm{with}\;r\in D,$$

with

$$a\left(r\right)=\left[\begin{array}{c} 2\partial_{x}{\hat{\varphi }}_{pq}^{\pm }\left(r\right)-2j\frac{\partial_{x}\left|{\hat{B}}_{1p}^{+}\left(r\right)\right|}{\left|{\hat{B}}_{1p}^{+}\left(r\right)\right|}\\ 2\partial_{y}{\hat{\varphi }}_{pq}^{\pm }\left(r\right)-2j\frac{\partial_{y}\left|{\hat{B}}_{1p}^{+}\left(r\right)\right|}{\left|{\hat{B}}_{1p}^{+}\left(r\right)\right|}\\ 2\partial_{z}{\hat{\varphi }}_{pq}^{\pm }\left(r\right)-2j\frac{\partial_{z}\left|{\hat{B}}_{1p}^{+}\left(r\right)\right|}{\left|{\hat{B}}_{1p}^{+}\left(r\right)\right|}\\ 1\\ 1 \end{array}\right],\;\;x\left(r\right)=\left[\begin{array}{c} \partial_{x}{\hat{\varphi }}_{q}^{-}\left(r\right)\\ \partial_{y}{\hat{\varphi }}_{q}^{-}\left(r\right)\\ \partial_{z}{\hat{\varphi }}_{q}^{-}\left(r\right)\\ {\left|\nabla {\hat{\varphi }}_{q}^{-}\left(r\right)\right|}^{2}-j\nabla^{2}{\hat{\varphi }}_{q}^{-}\left(r\right)\\ -k^{2}\left(r\right) \end{array}\right],$$

and

$$b\left(r\right)=\frac{\nabla^{2}{\hat{B}}_{1p}^{+;\mathrm{TRX}(p,q)}\left(r\right)}{{\hat{B}}_{1p}^{+;\mathrm{TRX}(p,q)}\left(r\right)},$$

Requiring that Equation (54) holds at *N* different locations with position vectors $r_{n}\in D$, n=1,2,…,N (e.g., with *N* the total number of pixels/voxels and $r_{n}$ the position vector of the center of the *n*th pixel/voxel), we obtain the set of equations $a^{T}\left(r_{n}\right)\;x\left(r_{n}\right)=b\left(r_{n}\right)$ for n=1,2,…,N, which can be written as an underdetermined system Ax=b, where A is an *N*-by-5N matrix given by

(55) $$A=\left[\begin{array}{cccc} a^{T}\left(r_{1}\right) & \; & & \\ \; & a^{T}\left(r_{2}\right) & \; & \\ \; & \; & \ddots & \\ \; & & \; & a^{T}\left(r_{N}\right) \end{array}\right]$$

and

(56) $$x={\left[\begin{array}{c} x^{T}\left(r_{1}\right),x^{T}\left(r_{2}\right),\ldots ,x^{T}\left(r_{N}\right) \end{array}\right]}^{T}\;\mathrm{and}\;b={\left[\begin{array}{c} b\left(r_{1}\right),b\left(r_{2}\right),\ldots b\left(r_{N}\right) \end{array}\right]}^{T}.$$

Since there are five unknowns associated with each point of interest $r_{n}$, at least five linearly independent transmit field measurements are carried out, producing the set of equations $A_{i}x=b_{i}$, i=1,2,…,I, where I≥5 is the total number of transmit field measurements. The total set of field equations can now be written as

(57) $$\left[\begin{array}{c} A_{1}\\ A_{2}\\ \vdots \\ A_{I} \end{array}\right]x=\left[\begin{array}{c} b_{1}\\ b_{2}\\ \vdots \\ b_{I} \end{array}\right]$$

and this square (I=5) or overdetermined (I>5) system is solved in the least-squares sense to obtain vector x. Finally, the EPs at location $r_{n}$ can be obtained by equating the fifth entry in $x(r_{n})$ to $-k^{2}\left(r_{n}\right)$.

This method requires no knowledge of the unavailable absolute transmit phase. However, since there are multiple unknowns for each point of interest, several independent transmit field measurements are required, which are typically only available on 7 T MRI systems, to derive a unique solution. The amount of required transmit fields can be reduced by extending the method with receive field measurements. The same procedure as described above can be carried out for the receive field in terms of measurable quantities, as presented in Equation (18), and, under the assumption of homogeneous proton density, a similar equation may be derived [40]. When more field maps are available, this last assumption can be prevented and the gradient and Laplacian of the proton density can also be determined [40]. LMT can be further generalized to also take the spatial variations of the tissue EPs into account, such that it becomes free from object and field assumptions. This, however, comes at the cost of increasing the number of unknowns and therefore requiring a larger amount of transmit and/or receive field maps [41].

### 4.4. Modified Dual-Excitation EPT

Modified dual-excitation EPT (MDE-EPT) uses Equation (33) as a starting point and it assumes that the gradient of the *z*-component of the magnetic flux density vanishes [28]. We then obtain

(58) $$\nabla^{2}{\hat{B}}_{1}^{+}=f\cdot \nabla {\hat{B}}_{1}^{+}-k^{2}{\hat{B}}_{1}^{+}=4\partial^{-}{\hat{B}}_{1}^{+}g^{+}+g_{z}\partial_{z}{\hat{B}}_{1}^{+}-k^{2}{\hat{B}}_{1}^{+}.$$

This local equation is assumed to hold inside the object domain D and can be written as

(59) $$a^{T}\left(r\right)\;x\left(r\right)=b\left(r\right),\;r\in D,$$

where a(r) and x(r) are 3-by-1 vectors given by

$$a\left(r\right)=\left[\begin{array}{c} \partial^{-}{\hat{B}}_{1}^{+}\left(r\right)\\ \partial_{z}{\hat{B}}_{1}^{+}\left(r\right)\\ {\hat{B}}_{1}^{+}\left(r\right) \end{array}\right]\;\mathrm{and}\;x\left(r\right)=\left[\begin{array}{c} 4g^{+}\left(r\right)\\ g_{z}\left(r\right)\\ -k^{2}\left(r\right) \end{array}\right]$$

and $b\left(r\right)=\nabla^{2}{\hat{B}}_{1}^{+}\left(r\right)$. Requiring that Equation (59) holds at *N* different locations with position vectors $r_{n}\in D$, n=1,2,…,N, leading to a system of equations Ax=b, where the *N*-by-3N matrix A, the 3N-by-1 vector x and the *N*-by-1 vector b are of similar form as in LMT (cf. Equations (55) and (56)). Since there are three unknowns ($g^{+}\left(r_{n}\right)$, $g_{z}\left(r_{n}\right)$, and $-k^{2}\left(r_{n}\right)$) associated with each point of interest $r_{n}$, at least three linearly independent transmit field measurements are carried out, producing the set of equations $A_{i}x=b_{i}$, i=1,2,…,I, where I≥3 is the total number of transmit field measurements. The total set of field equations can now again be written as Equation (57) and this square (I=3) or overdetermined (I>3) system is solved in the least-squares sense to obtain vector x. Finally, the EPs at location $r_{n}$ can be obtained by equating the third entry in $x(r_{n})$ to $-k^{2}\left(r_{n}\right)$.

This approach does not require homogeneity of the object, which allows for improved tissue boundary reconstructions. However, since there are three unknowns, at least three independent transmit fields are required. Additionally, the method is restricted to regions with spatially invariant *z*-component of the magnetic field. Note that the original form, dual-excitation EPT, assumed knowledge of the unavailable *x*- and *y*-components of the magnetic fields [42] and therefore required only two linearly independent excitations/measurements to determine the EP maps. MDE-EPT, however, can be extended by including Equation (39), again under the assumption of vanishing gradient of the *z*-component of the magnetic field, into the system of equations. The name dual-excitation is then again justified, in the sense that two independent excitations result in four equations if both the transmit and receive fields are acquired [43].

### 4.5. Gradient-Based EPT

Gradient-based EPT (G-EPT) continues from Equation (58) and writes it in terms of absolute and relative transmit phases with respect to a reference element, as presented in Equation (17) [44,45,46,47,48]. We then obtain

(60) $$\begin{array}{cc} \nabla^{2}{\hat{B}}_{1p}^{+;\mathrm{rel}(p,r)} & =-2j\nabla {\hat{B}}_{1p}^{+;\mathrm{rel}(p,r)}\cdot \nabla {\hat{\varphi }}_{r}^{+}-{\hat{B}}_{1p}^{+;\mathrm{rel}(p,r)}\left(-|\nabla {\hat{\varphi }}_{r}^{+}{|}^{2}+j\nabla^{2}{\hat{\varphi }}_{r}^{+}\right)\\ \; & +4\left(\partial^{-}{\hat{B}}_{1p}^{+;\mathrm{rel}(p,r)}+j{\hat{B}}_{1p}^{+;\mathrm{rel}(p,r)}\partial^{-}{\hat{\varphi }}_{r}^{+}\right)g^{+}\\ \; & +\left(\partial_{z}{\hat{B}}_{1p}^{+;\mathrm{rel}(p,r)}+j{\hat{B}}_{1p}^{+;\mathrm{rel}(p,r)}\partial_{z}{\hat{\varphi }}_{r}^{+}\right)g_{z}-k^{2}{\hat{B}}_{1p}^{+;\mathrm{rel}(p,r)}, \end{array}$$

with ${\hat{\varphi }}_{r}^{+}$ the unknown absolute transmit phase of reference channel *r*. First, the gradient $g^{+}$ is determined. Similar to LMT and MDE-EPT, the above equation is written in the form

(61) $$a^{T}\left(r\right)x\left(r\right)=b\left(r\right)\;\mathrm{with}\;r\in D,$$

where

$$a\left(r\right)=\left[\begin{array}{c} {\hat{B}}_{1p}^{+;\mathrm{rel}(p,r)}\left(r\right)\\ \partial_{x}{\hat{B}}_{1p}^{+;\mathrm{rel}(p,r)}\left(r\right)\\ \partial_{y}{\hat{B}}_{1p}^{+;\mathrm{rel}(p,r)}\left(r\right)\\ \partial_{z}{\hat{B}}_{1p}^{+;\mathrm{rel}(p,r)}\left(r\right) \end{array}\right],$$

$$x\left(r\right)=\left[\begin{array}{c} |\nabla {\hat{\varphi }}_{r}^{+}{\left(r\right)|}^{2}-j\nabla^{2}{\hat{\varphi }}_{r}^{+}\left(r\right)+4j\partial^{-}{\hat{\varphi }}_{r}^{+}\left(r\right)g^{+}\left(r\right)+j\partial_{z}{\hat{\varphi }}_{r}^{+}\left(r\right)g_{z}\left(r\right)-k^{2}\left(r\right)\\ -2j\partial_{x}{\hat{\varphi }}_{r}^{+}\left(r\right)+4g^{+}\left(r\right)\\ -2j\partial_{y}{\hat{\varphi }}_{r}^{+}\left(r\right)-4jg^{+}\left(r\right)\\ -2j\partial_{z}{\hat{\varphi }}_{r}^{+}\left(r\right)+g_{z}\left(r\right) \end{array}\right],$$

and $b\left(r\right)=\nabla^{2}{\hat{B}}_{1p}^{+;\mathrm{rel}(p,r)}\left(r\right)$. Equation (61) is required to hold at *N* different locations of interest with position vectors $r_{n}\in D$, n=1,2,…,N, leading to a system of equations Ax=b, where the *N*-by-4N matrix A, the 4N-by-1 vector x and the *N*-by-1 vector b are of a similar form as in LMT and MDE-EPT (cf. Equations (55) and (56)).

Since there are four unknowns associated with each point of interest (the elements of vector $x(r_{n})$), at least four linearly independent transmit field measurements are carried out and these produce the set of equations $A_{i}x=b_{i}$, i=1,2,…,I, with I≥4 the total number of transmit field measurements. The total set of equations can now again be written as in Equation (57) and this square (I=4) or overdetermined (I>4) system is solved in the least-squares sense to obtain vector x. From this vector, $g^{+}\left(r_{n}\right)$ can be determined from the second or third entry of $x(r_{n})$.

Second, the gradient is integrated using the definition $g^{+}=\partial^{+}\ln\left(\eta \right)$ and an additional least-squares minimization process, where seed points (point belonging to a subdomain of the object domain where the EPs are known) are used to obtain absolute EP maps.

The additional integration step in G-EPT acts as a low-pass filter and makes the approach relatively robust to noise. Additionally, using the relative phase has the benefit that influences of receive field, chemical shift, magnetic susceptibility and eddy currents on the phase are mitigated. The method, however, requires multiple transmit elements as well as knowledge of seed points to derive absolute EP maps. The seed points can be derived by surrounding the object with a gel with known EPs, (dubbed boundary informed G-EPT) [48]. Additionally, since the transverse gradients of the absolute phase of the reference channel can also be derived from x(2) and x(3) in the first step of G-EPT, the seed points can be selected in an automated fashion by using the Helmholtz-based EPT approach in homogeneous regions (dubbed automated G-EPT) [47].

### 4.6. Convection–Reaction EPT

Convection–reaction EPT (CR-EPT) [49,50,51] assumes that the ${\hat{B}}_{1}^{+}$ field is known and solves the generalized Helmholtz equation in convection–reaction form (Equation (36)), for convenience repeated here,

(62) $$u\nabla^{2}{\hat{B}}_{1}^{+}+\beta^{+}\cdot \nabla u-\zeta {\hat{B}}_{1}^{+}=0,$$

in a least-squares sense for the inverse admittance parameter *u* under the assumption of invariance of the *z*-component of the magnetic flux density in the convective field (Equation (35)), and derives the tissue parameters as

(63) σ=Re1u,andε=1ωIm1u.

This method is again not restricted to regions with homogeneous tissue structures, does not require seed points and does not require a multi-element array. However, the absolute transmit phase is again required, which is not directly available from measurements but can be accurately estimated for many cases as half the transceive phase. Furthermore, the method is restricted to regions with spatially invariant *z*-component of the magnetic field. Additionally, the method suffers from a reconstruction artifact in the region with low convective field.

#### 4.6.1. Phase-Only Convection–Reaction Conductivity Mapping

Phase-only convection–reaction conductivity mapping (PCR-CM) [52] simplifies the generalized Helmholtz equation in convection–reaction form (Equation (36)) by dividing it by ${\hat{B}}_{1}^{+}$ and assuming $\nabla \left|{\hat{B}}_{1}^{+}\right|=0$ and $\nabla {\hat{B}}_{z}=0$, which gives

(64) $$u\left(-{\left|\nabla {\hat{\varphi }}^{+}\right|}^{2}+j\nabla^{2}{\hat{\varphi }}^{+}\right)+{\tilde{\beta }}^{+}\cdot \nabla u-\zeta =0,$$

with

$${\tilde{\beta }}^{+}=2j\partial^{-}{\hat{\varphi }}^{+}i_{x}-2\partial^{-}{\hat{\varphi }}^{+}i_{y}+j\partial_{z}{\hat{\varphi }}^{+}i_{z}.$$

The same procedure can be performed for the generalized Helmholtz equation in convection–reaction form in terms of receive fields (Equation (40)), which yields

(65) $$u\left(-{\left|\nabla {\hat{\varphi }}^{-}\right|}^{2}-j\nabla^{2}{\hat{\varphi }}^{-}\right)+{\tilde{\beta }}^{-}\cdot \nabla u-\zeta =0,$$

with

$${\tilde{\beta }}^{-}=-2j\partial^{+}{\hat{\varphi }}^{-}i_{x}-2\partial^{+}{\hat{\varphi }}^{-}i_{y}-j\partial_{z}{\hat{\varphi }}^{-}i_{z}.$$

The addition of these two equations gives

(66) $$u\left(-{\left|\nabla {\hat{\varphi }}^{\pm }\right|}^{2}-2\nabla {\hat{\varphi }}^{+}\cdot \nabla {\hat{\varphi }}^{-}+j\nabla^{2}{\hat{\varphi }}^{\pm }\right)+{\tilde{\beta }}^{\pm }\cdot \nabla u-2\zeta =0,$$

with

$${\tilde{\beta }}^{\pm }=\left[\partial_{y}\left({\hat{\varphi }}^{+}+{\hat{\varphi }}^{-}\right)+j\partial_{x}{\hat{\varphi }}^{\pm }\right]i_{x}-\left[\partial_{x}\left({\hat{\varphi }}^{+}+{\hat{\varphi }}^{-}\right)+j\partial_{y}{\hat{\varphi }}^{\pm }\right]i_{y}+j\partial_{z}{\hat{\varphi }}^{\pm }.$$

In the case that Imβ˜±·Re∇u>>Reβ˜±·Im∇u, the imaginary part of this equation can be written as a convection–reaction equation in terms of the resistivity $\rho =\sigma^{-1}$ as

(67) $$\rho \nabla^{2}{\hat{\varphi }}^{\pm }+\nabla {\hat{\varphi }}^{\pm }\cdot \nabla \rho -2\omega \mu_{0}=0,$$

which can be solved in a least-squares sense. This equation is in the form of a convection–diffusion–reaction equation with zero diffusion term. A diffusion term would act as a low-pass filter and increases numerical stability of the approach. To suppress spurious oscillations, an artificial diffusion term $c\nabla^{2}\rho$ is typically added to the fundamental equation, where *c* is an empirically determined constant diffusion coefficient. The conductivity σ is finally retrieved as the inverse of the resistivity ρ.

This method can be seen as a generalized version of phase-only Helmholtz-based EPT implementation as discussed in Section 4.2, which allows for large spatial variations of the tissue conductivity. However, the method has an increased computational complexity and the required assumptions do not hold for high field strengths.

### 4.7. Transverse EPT

Transverse EPT (T-EPT) [53] assumes that the RF field has a so-called E-polarized field structure within a certain transverse plane, by which we mean that longitudinal variations of the transverse electric field and the longitudinal variation of the magnetic field essentially vanish within this plane ($\partial_{z}{\hat{E}}_{x}=\partial_{z}{\hat{E}}_{y}=0$, and $\partial_{z}{\hat{B}}_{z}=0$ for z=constant). Usually, the plane z=constant is taken to be the midplane of a birdcage coil, since it has been observed that the RF field has an approximate E-polarized field structure within this midplane [54]. Note that, for two-dimensional configurations with no spatial variations in the *z*-direction and a *z*-directed external electric current source, the E-polarized field structure is exact.

Taking the E-polarized field assumption into account, it follows from Maxwell’s equations that (cf. Equations (24) and (37))

(68) $$\frac{4}{j\mu_{0}}\partial^{-}{\hat{B}}_{1}^{+}=\eta {\hat{E}}_{z}\;\mathrm{and}\;{\hat{B}}_{1}^{+}=\frac{1}{\omega }\partial^{+}{\hat{E}}_{z},\;\mathrm{within}\;\mathrm{the}\;\mathrm{plane}\;z=\mathrm{constant}.$$

In T-EPT, these two equations are combined into a single normalized functional given by

(69) $$F\left({\hat{E}}_{z},\eta \right)=\frac{{∥\frac{4}{j\mu_{0}}\partial^{-}{\hat{B}}_{1}^{+}-\eta {\hat{E}}_{z}∥}_{D}^{2}}{{∥\frac{4}{j\mu_{0}}\partial^{-}{\hat{B}}_{1}^{+}∥}_{D}^{2}}+\frac{{∥{\hat{B}}_{1}^{+}-\frac{1}{\omega }\partial^{+}{\hat{E}}_{z}∥}_{D}^{2}}{{∥{\hat{B}}_{1}^{+}∥}_{D}^{2}},$$

where ${∥\cdot ∥}_{D}$ is an $L^{2}$-norm defined on D. Ths functional is iteratively minimized in an alternate fashion using conjugate-gradient-type update formulas for ${\hat{E}}_{z}$, followed by a direct update of η. This two-step update procedure is repeated until convergence or a maximum number of iterations is reached. Finally, the conductivity and permittivity reconstructions follow from the reconstructed admittance η as

(70) σ=Reηandε=1ωImη.

In the remainder of this manuscript, these multi-step inversion methods are summarized in a listing (see Listing 1 for the update process of T-EPT).

This method has no second-order but only first-order derivatives that act on the measurement data, increasing noise robustness. Additionally, the method computes the *z*-component of the electric field strength, which can be helpful in SAR computations. However, the method is restricted to regions where the RF field is approximately E-polarized, such as in the midplane of a birdcage RF coil.

**Listing 1.** Transverse EPT (T-EPT).
Given initial guesses ${\tilde{\eta }}^{\left[0\right]}$ for the admittance and ${\tilde{E}}_{z}^{\left[0\right]}$ for the electric field strengthFor n=1,2,…
(a)Fix the admittance ${\tilde{\eta }}^{\left[n-1\right]}$ and update the electric field strength according to the update formula
$${\tilde{E}}_{z}^{\left[n\right]}={\tilde{E}}_{z}^{\left[n-1\right]}+\alpha^{\left[n\right]}v^{\left[n\right]}$$
where α are the update coefficients and *v* the Polak-Ribière update directions [55].(b)Update the admittance according to
${\tilde{\eta }}^{\left[n\right]}=\frac{4}{j\mu_{0}}\frac{{\tilde{E}}_{z}^{[n]*}\partial^{-}{\hat{B}}_{1}^{+}}{{\left|{\tilde{E}}_{z}^{\left[n\right]}\right|}^{2}}.$(c)Stop if objective function is smaller than user specified tolerance level, or if maximum number of iterations has been reached.End


### 4.8. First-Order Induced-Current EPT

First-order induced-current EPT (foIC-EPT) considers E-polarized RF fields and thus assumes that the electric field strength is mainly directed in the longitudinal *z*-direction [56]. For E-polarized fields, we have (cf. Equations (37) and (44))

(71) $$\eta {\hat{E}}_{z}=\frac{4}{j\mu_{0}}\partial^{-}{\hat{B}}_{1}^{+}\;\mathrm{and}\;{\hat{E}}_{z}^{\mathrm{sca}}=-\zeta G_{A}\left\{\eta {\hat{E}}_{z}\right\}-k_{0}^{2}G_{A}\left\{{\hat{E}}_{z}\right\},$$

where we introduce the vector potential operator

$$G_{A}\left\{x\right\}\left(r\right)=\eta_{0}^{-1}\int_{r^{\prime }\in D}\hat{G}\left(r-r^{\prime }\right)x\left(r^{\prime }\right)\;dV.$$

Combining these two equations, together with the linearity of Maxwell’s equations (see Equation (42)), gives

(72) $${\hat{E}}_{z}^{\mathrm{inc}}-4\omega G_{A}\left\{\partial^{-}{\hat{B}}_{1}^{+}\right\}={\hat{E}}_{z}+k_{0}^{2}G_{A}\left\{{\hat{E}}_{z}\right\},$$

which can be solved for the *z*-component of the electric field strength, if its incident component is known. Finally, the conductivity and permittivity can be derived via

(73) σ=Re4jμ0E^z∗∂−B^1+|E^z|2andε=1ωIm4jμ0E^z∗∂−B^1+|E^z|2.

This approach has only first-order derivatives that act on the measured transmit field data, and an integral formulation for the electric field strength determination, making the method robust to noise. However, the method is again restricted to a region with an E-polarized field structure. Additionally, the method requires knowledge of the incident field, which cannot be measured directly. Incident fields are typically estimated from a simulation setup or from a reference scan of a phantom with known EPs. Note that the formulation is presented as a three-dimensional problem, but, in the case of an E-polarized field structure, it can be simplified to a two-dimensional setting. Cauchy-based EPT shares a lot of similarities with foIC-EPT, but the electric field strength is derived via a Cauchy integral which allows for the computation of the EPs in a direct manner through complex analysis [57,58,59,60].

### 4.9. Variational Born Iterative Method EPT

The variational Born iterative method EPT (VBIM-EPT) is a volumetric integral method that iteratively updates the tissue parameters based on improved estimations of the transmit field by solving forward and inverse problems [61,62]. Given knowledge of the incident fields and an initial estimation for the contrast function, the electric field strength is derived from (cf. Equations (42) and (44))

(74) $${\hat{E}}^{\mathrm{inc}}\left(r\right)=\hat{E}\left(r\right)-\left(k_{0}^{2}+\nabla \nabla \cdot \right)\int_{r^{\prime }\in D}\hat{G}\left(r-r^{\prime }\right)\chi \left(r^{\prime }\right)\hat{E}\left(r^{\prime }\right)\;dV,$$

with $\chi =\left(\eta -\eta_{0}\right)\eta_{0}^{-1}$. Equation (74) is a forward problem. Based on the derived electric field strength and the estimated contrast function, an estimate of the scattered part of the transmit field is computed as (cf. Equation (45))

(75) $${\tilde{B}}_{1}^{+;\mathrm{sca}}\left(r\right)=\frac{\omega }{c_{0}^{2}}\tilde{\nabla }\cdot \int_{r^{\prime }\in D}\hat{G}\left(r-r^{\prime }\right)\chi \left(r^{\prime }\right)\hat{E}\left(r^{\prime }\right)\;dV,$$

and the residual $\delta {\hat{B}}_{1}^{+;\mathrm{sca}}$ is derived according to $\delta {\hat{B}}_{1}^{+;\mathrm{sca}}={\hat{B}}_{1}^{+}-{\hat{B}}_{1}^{+;\mathrm{inc}}-{\tilde{B}}_{1}^{+;\mathrm{sca}}$. The residual in the contrast function is then determined by solving

(76) $$\delta {\hat{B}}_{1}^{+;\mathrm{sca}}\left(r\right)=\frac{\omega }{c_{0}^{2}}\tilde{\nabla }\cdot \int_{r^{\prime }\in D}\hat{G}\left(r-r^{\prime }\right)\delta \chi \left(r^{\prime }\right)\hat{E}\left(r^{\prime }\right)\;dV$$

for δχ, which is an inverse problem. The contrast function is then updated as $\chi^{\left[n+1\right]}=\chi^{\left[n\right]}+\delta \chi$. Based on this new estimation of the contrast function, the procedure is repeated until a convergence criterion has been reached (see Listing 2). Finally, the conductivity and permittivity maps are derived via

(77) σ=−ωε0Imχ,andε=ε0Reχ+1

This method does not apply any derivatives on the measured transmit field. Instead, it makes use of an integral formulation, making the method noise robust. However, the method requires knowledge of the incident fields, and solving the forward and inverse problems iteratively is computationally prohibitively expensive.

**Listing 2.** Variational Born Iterative Method-EPT (VBIM-EPT).
Given initial guesses ${\tilde{\chi }}^{\left[0\right]}$ for the contrast functionFor n=1,2,…
(a)Fix the contrast function to ${\tilde{\chi }}^{\left[n-1\right]}$ and determine the electric field strength ${\tilde{E}}^{\left[n\right]}$ by solving Equation (74) for $\hat{E}$ (solve the forward problem).(b)Knowing contrast function ${\tilde{\chi }}^{\left[n-1\right]}$ and corresponding electric field strength ${\tilde{E}}^{\left[n\right]}$, compute the scattered magnetic flux density ${\tilde{B}}_{1}^{+;\mathrm{sca};[n]}$ according to Equation (75).(c)Compute the residual $\delta {\hat{B}}_{1}^{+;\mathrm{sca};[n]}$ according to
$$\delta {\hat{B}}_{1}^{+;\mathrm{sca};[n]}={\hat{B}}_{1}^{+}-{\hat{B}}_{1}^{+;\mathrm{inc}}-{\tilde{B}}_{1}^{+;\mathrm{sca};[n]}.$$(d)Fix the data residual to $\delta {\hat{B}}_{1}^{+;\mathrm{sca};[n]}$ and the electric field strength to ${\tilde{E}}^{\left[n\right]}$ and determine the contrast residual $\delta \chi^{\left[n\right]}$ by solving Equation (76) for δχ (solve the inverse problem).(e)Update the contrast function according to the update formula
$${\tilde{\chi }}^{\left[n\right]}={\tilde{\chi }}^{\left[n-1\right]}+\delta \chi^{\left[n\right]}.$$(f)Stop if $\delta {\hat{B}}_{1}^{+;\mathrm{sca};[n]}$ is smaller than user specified tolerance level, or if maximum number of iterations has been reached.End


### 4.10. Global Maxwell Tomography

Global Maxwell tomography (GMT) is a volumetric integral method that iteratively updates the tissue parameters based on improved estimations of the transmit field by solving a forward problem and minimizing an objective function [63,64,65]. GMT makes use of the identity [66]

$$\left(k_{0}^{2}+\nabla \nabla \cdot \right)\int_{r^{\prime }\in D}\hat{G}\left(r-r^{\prime }\right)\hat{w}\left(r^{\prime }\right)\;dV=\nabla \times \nabla \times \int_{r^{\prime }\in D}\hat{G}\left(r-r^{\prime }\right)\hat{w}\left(r^{\prime }\right)\;dV-\hat{w},\;r\in D,$$

and transforms the electric field integral representation of Equation (74) into a current density volume integral representation, given by

(78) $$\eta_{0}\chi \left(r\right){\hat{E}}^{\mathrm{inc}}\left(r\right)=\left(1+\chi \left(r\right)\right){\hat{J}}^{\mathrm{sca}}\left(r\right)-\chi \left(r\right)\nabla \times \nabla \times \eta_{0}^{-1}\int_{r^{\prime }\in D}\hat{G}\left(r-r^{\prime }\right){\hat{J}}^{\mathrm{sca}}\left(r\right)\;dV.$$

Given knowledge of the incident field and an initial contrast function, this equation is solved for the scattered current density distribution (a forward problem), which is used to estimate the scattered component of the transmit field ${\tilde{B}}_{1}^{+;\mathrm{sca}}$ via Equation (45). An objective function is introduced

(79) $$F\left(\chi \right)=\frac{{∥{\hat{B}}_{1}^{+;\mathrm{sca}}-{\tilde{B}}_{1}^{+;\mathrm{sca}}\left({\hat{J}}^{\mathrm{sca}}\left(\chi \right)\right)∥}_{D}^{2}}{{∥{\hat{B}}_{1}^{+;\mathrm{sca}}∥}_{D}^{2}}.$$

Based on its gradient with respect to χ the contrast function is updated. This process is iterated until a convergence criterion has been reached (see Listing 3). Finally, the EPs are derived from the contrast function via Equation (77).

This method is similar to VBIM-EPT, but removes the inverse problem in every iteration. Even though a computational expensive inverse problem is removed, the method remains computationally expensive since the gradient updates typically require a large amount of iterations. The presented formulation still requires knowledge of the absolute transmit phase; however, this has been addressed by reformulating the objective function to only consider the magnitude of the transmit field or, in the case of a multi-element transmit system, by reformulating it in terms of magnitude and relative phases [64,65].

**Listing 3.** Global Maxwell Tomography (GMT).
Given initial guess ${\tilde{\chi }}^{\left[0\right]}$ for the contrast functionFor n=1,2,…
(a)Fix the contrast function to ${\tilde{\chi }}^{\left[n-1\right]}$ and determine the scattered current density distribution ${\tilde{J}}^{\mathrm{sca};[n]}$ by solving Equation (78) for ${\hat{J}}^{\mathrm{sca}}$ (solve the forward problem).(b)Fix the scattered current density distribution to ${\tilde{J}}^{\mathrm{sca};[n]}$ and update the contrast function according to the update formula
$${\tilde{\chi }}^{\left[n\right]}={\tilde{\chi }}^{\left[n-1\right]}+\beta^{\left[n\right]}d^{\left[n\right]}.$$
where β are the update coefficients and *d* the Polak-Ribière update directions [55](c)Stop if objective function of Equation (79) is smaller than user specified tolerance level, or if maximum number of iterations has been reached.End


### 4.11. Contrast Source Inversion EPT

Contrast-Source Inversion EPT (CSI-EPT) formulates the inversion problem as a purely optimization problem in which a single functional is iteratively minimized [67,68]. CSI-EPT combines the multiplication of the contrast function and the electric field strength into a single variable, the so-called contrast source $\hat{w}=\chi \hat{E}$. The scattered electric field strength is then given by (cf. Equation (44))

$${\hat{E}}^{\mathrm{sca}}\left(r\right)=\left(k_{0}^{2}+\nabla \nabla \cdot \right)\int_{r^{\prime }\in D}\hat{G}\left(r-r^{\prime }\right)\hat{w}\left(r^{\prime }\right)G_{E}\left\{\hat{w}\right\}\left(r\right),$$

and the scattered transmit field operator is then given by (cf. Equation (45))

$${\hat{B}}_{1}^{+;\mathrm{sca}}\left(r\right)=\frac{\omega }{c_{0}^{2}}\tilde{\nabla }\cdot \int_{r^{\prime }\in D}\hat{G}\left(r-r^{\prime }\right)\hat{w}\left(r^{\prime }\right)G_{B}\left\{\hat{w}\right\}\left(r\right),$$

which are used to set up an objective functional (cf. Equation (74))

(80) $$F\left(\hat{w},\chi \right)=\frac{{∥\chi {\hat{E}}^{\mathrm{inc}}-\hat{w}+\chi G_{E}\left\{\hat{w}\right\}∥}_{D}^{2}}{{∥\chi {\hat{E}}^{\mathrm{inc}}∥}_{D}^{2}}+\frac{{∥{\hat{B}}_{1}^{+;\mathrm{sca}}-G_{B}\left\{\hat{w}\right\}∥}_{D}^{2}}{{∥{\hat{B}}_{1}^{+;\mathrm{sca}}∥}_{D}^{2}},$$

which is minimized in a two-step “fix-one-minimize-for-the-other” update process. First, the contrast function is fixed and the contrast source is updated from the gradient of the cost function with respect to $\hat{w}$. Once the contrast source is updated, the electric field strength is calculated as

$$\hat{E}={\hat{E}}^{\mathrm{inc}}+G_{E}\left\{\hat{w}\right\},$$

and the contrast function is updated by solving the least-squares problem ${∥\tilde{\chi }\tilde{E}-\tilde{w}∥}_{D}^{2}$, which gives

$$\chi =\frac{\hat{w}\cdot {\hat{E}}^{*}}{{\left|\hat{E}\right|}^{2}},$$

or by fixing the contrast source and updating the contrast function from the gradient of the cost functional with respect to χ. This two-step update procedure is iteratively repeated until a stopping criterion has been reached (see Listing 4). Finally, the tissue parameters are derived from the contrast function via Equation (77).

This approach only applies fast forward computations and does not have to solve any forward problem as in VBIM-EPT or GMT. However, since the approach typically still requires a lot of iterations, the method remains time consuming compared to direct methods such as H-EPT. Additionally, the transmit phase remains required, which can only be accurately approximated in specific cases. Naive [69,70,71,72], two-dimensional [73,74,75,76,77,78], magnitude-based [79] and segmented [80,81] implementations have been proposed to improve for example convergence or applicability.

**Listing 4.** Contrast Source Inversion-EPT (CSI-EPT).
Given initial guesses ${\tilde{\chi }}^{\left[0\right]}$ and ${\tilde{w}}^{\left[0\right]}$ for the contrast function and contrast source, respectivelyFor n=1,2,…
(a)Fix the contrast function to ${\tilde{\chi }}^{\left[n-1\right]}$ and update the contrast source according to the update formula [67]
$${\tilde{w}}^{\left[n\right]}={\tilde{w}}^{\left[n-1\right]}+\alpha^{\left[n\right]}v^{\left[n\right]}.$$(b)Compute the corresponding electric field strength ${\hat{E}}^{\left[n\right]}$ according to
${\tilde{E}}^{\left[n\right]}={\hat{E}}^{\mathrm{inc}}+G_{E}\left\{{\tilde{w}}^{\left[n\right]}\right\}.$(c)Compute the contrast function according to
$$\tilde{\chi }=\frac{\tilde{w}\cdot {\tilde{E}}^{*}}{{\left|\tilde{E}\right|}^{2}},$$
or fix the contrast source to ${\tilde{w}}^{\left[n\right]}$ and update the contrast function according to the update formula [67]
${\tilde{\chi }}^{\left[n\right]}={\tilde{\chi }}^{\left[n-1\right]}+\beta^{\left[n\right]}d^{\left[n\right]}.$(d)Stop if objective function of Equation (80) is smaller than user specified tolerance level, or if maximum number of iterations has been reached.End


## 5. EPT Methods Not Requiring Transmit Field Mapping

The previously discussed EPT approaches can be extended with receive fields. However, there are also methods that do not require transmit fields. The followin EPT approaches are discussed in this section:

- Section 5.1: Single-acquisition EPT (SA-EPT)
- Section 5.2: Image-based EPT (I-EPT)

### 5.1. Single-Acquisition EPT

Single-acquisition EPT (SA-EPT) [82] rewrites the Helmholtz equation for the receive field (Equation (41)) as

(81) $$\begin{array}{cc} \frac{\nabla^{2}{\hat{B}}_{1}^{-;*}}{{\hat{B}}_{1}^{-;*}} & =\frac{\nabla \cdot \left({\hat{B}}_{1}^{-;*}\frac{\nabla {\hat{B}}_{1}^{-;*}}{{\hat{B}}_{1}^{-;*}}\right)}{{\hat{B}}_{1}^{-;*}},\\ \; & =\frac{\nabla {\hat{B}}_{1}^{-;*}}{{\hat{B}}_{1}^{-;*}}\cdot \frac{\nabla {\hat{B}}_{1}^{-;*}}{{\hat{B}}_{1}^{-;*}}+\nabla \cdot \left(\frac{\nabla {\hat{B}}_{1}^{-;*}}{{\hat{B}}_{1}^{-;*}}\right)=-k^{2}, \end{array}$$

which shows that knowledge of the rate of change of the field is sufficient to derive the EPs. By introducing the receive field of channel *q* relative to reference channel *r* as

(82) $${\hat{B}}_{1qr}^{-;*}=\frac{{\hat{B}}_{1q}^{-;*}}{{\hat{B}}_{1r}^{-;*}},$$

we obtain through the Laplacian of the relative receive field [82]

(83) $$\begin{array}{cc} \nabla^{2}{\hat{B}}_{1qr}^{-;*} & =\frac{{\hat{B}}_{1q}^{-;*}}{{\hat{B}}_{1r}^{-;*}}\left(\frac{\nabla^{2}{\hat{B}}_{1q}^{-;*}}{{\hat{B}}_{1q}^{-;*}}-\frac{\nabla^{2}{\hat{B}}_{1r}^{-;*}}{{\hat{B}}_{1r}^{-;*}}\right)-2\nabla {\hat{B}}_{1qr}^{-;*}\cdot \frac{\nabla {\hat{B}}_{1r}^{-;*}}{{\hat{B}}_{1r}^{-;*}},\\ \; & =-2\nabla {\hat{B}}_{1qr}^{-;*}\cdot \frac{\nabla {\hat{B}}_{1r}^{-;*}}{{\hat{B}}_{1r}^{-;*}}, \end{array}$$

since each element measures the same EPs. This local equation is assumed to hold inside the object domain D and can be written as

(84) $$a^{T}\left(r\right)\;x\left(r\right)=b\left(r\right),\;r\in D,$$

where a(r) and x(r) are 3-by-1 vectors given by

$$a\left(r\right)=\left[\begin{array}{c} -2\partial_{x}{\hat{B}}_{1qr}^{-;*}\left(r\right)\\ -2\partial_{y}{\hat{B}}_{1qr}^{-;*}\left(r\right)\\ -2\partial_{z}{\hat{B}}_{1qr}^{-;*}\left(r\right) \end{array}\right]\;\mathrm{and}\;x\left(r\right)=\left[\begin{array}{c} \frac{\partial_{x}{\hat{B}}_{1r}^{-;*}}{{\hat{B}}_{1r}^{-;*}}\left(r\right)\\ \frac{\partial_{x}{\hat{B}}_{1r}^{-;*}}{{\hat{B}}_{1r}^{-;*}}\left(r\right)\\ \frac{\partial_{x}{\hat{B}}_{1r}^{-;*}}{{\hat{B}}_{1r}^{-;*}}\left(r\right) \end{array}\right]$$

and $b\left(r\right)=\nabla^{2}{\hat{B}}_{1qr}^{-;*}\left(r\right)$. Equation (84) is required to hold at *N* different locations of interest with position vectors $r_{n}\in D$, n=1,2,…,N, leading to a system of equations Ax=b, where the *N*-by-3N matrix A, the 3N-by-1 vector x and the *N*-by-1 vector b are of a similar form as in LMT, MDE-EPT and G-EPT (cf. Equations (55) and (56)). Once the $\frac{\nabla {\hat{B}}_{1}^{-;*}}{{\hat{B}}_{1}^{-;*}}$ term is obtained, the tissue parameters can be determined from Equation (81).

This method does not require absolute transmit field data, but relies only on relative receive fields which are directly available. This results in the elimination of specific artifacts, since common terms for the different elements can be eliminated. These receive fields can be derived from a single acquisition; however, this requires a multi-element array with a minimum of four receive elements, since at least three linearly independent relative receive fields are required to determine a unique solution. Additionally, since the gradient term is derived in a minimization process based on second-order derivatives and an additional divergence is applied on the gradient term, the method relies on third-order derivatives giving strong dependence on the SNR of the images.

### 5.2. Image-Based EPT

Image-based EPT (I-EPT) uses the acquired MR image directly for the reconstruction of the tissue parameters. For a low flip angle $\alpha =\gamma \tau \left|{\hat{B}}_{1}^{+}\right|$, we have sinα≈α, thus the MR image for any low-flip-angle sequence is essentially given by (cf. Equation (15))

(85) $$I=\varrho_{0}\gamma \tau {\hat{B}}_{1}^{+}{\hat{B}}_{1}^{-;*}.$$

In I-EPT, information from this image is used, instead of estimated transmit or receive fields. The relevant equation applied to this image is derived by multiplying Equation (29) with ${\hat{B}}_{1}^{-;*}$ and Equation (41) with ${\hat{B}}_{1}^{+}$ and adding them together, which gives

(86) $$\nabla^{2}\left({\hat{B}}_{1}^{+}{\hat{B}}_{1}^{-;*}\right)+2k^{2}{\hat{B}}_{1}^{+}{\hat{B}}_{1}^{-;*}-2\nabla {\hat{B}}_{1}^{+}\cdot \nabla {\hat{B}}_{1}^{-;*}=0.$$

By taking ${\hat{B}}_{1}^{+}{\hat{B}}_{1}^{-;*}={\left(\sqrt{{\hat{B}}_{1}^{+}{\hat{B}}_{1}^{-;*}}\right)}^{2}$ and defining $a=\sqrt{{\hat{B}}_{1}^{+}{\hat{B}}_{1}^{-;*}}$ and $b=\sqrt{\frac{{\hat{B}}_{1}^{-;*}}{{\hat{B}}_{1}^{+}}}$, we obtain

$$\nabla^{2}a^{2}+2k^{2}a^{2}-2\nabla \frac{a}{b}\cdot \nabla \left(ab\right)=0,$$

and by using the product rule of the scalar Laplacian ($\nabla^{2}a^{2}=2a\nabla^{2}a+2\nabla a\cdot \nabla a$) and dividing by $2a^{2}$, we find

(87) $$\frac{\nabla^{2}a}{a}+k^{2}+\underset{\left(*\right)}{\underset{︸}{\frac{1}{a^{2}}\left(\nabla a\cdot \nabla a-\nabla \frac{a}{b}\cdot \nabla \left(ab\right)\right)}}=0.$$

The underbraced term denoted by (∗) is an error term that can be simplified to $\frac{1}{4}{\left|\nabla \ln\frac{{\hat{B}}_{1}^{-;*}}{{\hat{B}}_{1}^{+}}\right|}^{2}$ [83] and can be neglected when ${\hat{B}}_{1}^{+}$ and ${\hat{B}}_{1}^{-;*}$ are similar to each other. This results in the following Helmholtz equation

(88) $$\frac{\nabla^{2}\sqrt{{\hat{B}}_{1}^{+}{\hat{B}}_{1}^{-;*}}}{\sqrt{{\hat{B}}_{1}^{+}{\hat{B}}_{1}^{-;*}}}=-k^{2}.$$

Equation (88) remains valid when the ${\hat{B}}_{1}^{+}{\hat{B}}_{1}^{-;*}$ term is multiplied by a constant, since it drops out of the equation. The variables in front of the ${\hat{B}}_{1}^{+}{\hat{B}}_{1}^{-;*}$ term in Equation (88) are relatively constant throughout space in regions where the Helmholtz equation applies, and the image as described in Equation (85) can therefore be applied in Equation (88).

This method does not require the acquisition of transmit and receive fields, which results in reduced scan time and an increase in SNR, since the image SNR is greater than that of transmit or receive field maps. Additionally, in this formulation, the errors resulting from ${\hat{B}}_{1}^{+}$ and ${\hat{B}}_{1}^{-}$ differences are reduced to a first-order effect with respect to the difference compared to the conventional H-EPT method. A zero echo-time sequence has been proposed due to its immunity to eddy current and static magnetic field ($B_{0}$) inhomogeneity-induced phase changes, as well as its speed and SNR efficiency [83]. The method has also been proposed with a fast spin echo sequence together with a $T_{2}$ relaxation pattern between echoes to increase noise robustness [84]. A generalized image-based EPT form which includes the gradient of the EPs has also been proposed [85].

## 6. Data Driven Deep Learning Approaches for Solving Inverse Problems

Solving inverse problem by data-driven deep learning approaches is an emerging field with recent examples from the fields of gravitation [86], radioastronomy [87], medical imaging reconstruction [88] and electromagnetics [89,90]. The basic advantage of these data-driven approaches is that it allows insertion of more tailored a priori information about the specific inverse problem under study. A key aspect is the learning-based approach where during a training phase deep neural networks learn to perform a specific task in the inverse process by feeding them with many ground truth examples. After training the neural network, the inference is extremely fast, sometimes only a few seconds. This constitutes a clear computational gain over more conventional iterative approaches to solve inverse problems. Other advantages include a higher level of tolerance to noise on the input data and a higher flexibility on the required input data as neural networks can act as learned surrogate models.

### 6.1. Convolutional Neural Networks

The most popular for image processing and reconstruction are convolutional neural networks (CNN) [91,92,93]. These neural networks employ convolutions in their architecture whose kernels are optimized during training for their given task. The training phase is a very computationally intensive process involving the backpropagation of the errors during the training (in fact, a large scale optimization process itself) over the various weights between the nodes (often >1 million) in the network. This process takes place on a GPU and is facilitated by mature software packages that are able to exploit the parallel nature of the GPU in an efficient manner.

To obtain an idea on what information a trained CNN triggers, it is insightful to process an input image through the trained network and generate the output of the various units in the layers as images (so-called feature maps) comprising of local and global predictive information to perform the task it was trained for. Although CNNs are also heavily used for classification problems such as image segmentation, for use in the EPT reconstruction, only the regression task is relevant. In a regression CNN, where the famous U-NET [93] is a prime example, an encoder and the image information is processed by various convolutional kernels and pooling operations and fed through activation layers which constitute non-linear elements and are essential for the ability to learn. During the downsampling path, spatial contextual information is learnt. In regression problems, this is followed by the decoder where convolution and upsampling takes place to recover the spatial information of the desired output matrix size. In fully convolutional networks, the architecture solely employs operations such as convolution, pooling, activation and upsampling. Avoiding fully connected layers makes the inference much faster as fewer weights are needed, and the network can work regardless of the original image size. Skip connections short-circuiting corresponding layers in the encoder and decoder such as in the popular U-NET [93] are essential to recover fine-grained spatial information lost in the pooling or downsampling layers.

### 6.2. Deep Learning for EPT Reconstruction: Single Feedforward Approaches

An important distinction can be made between deep learning inverse approaches where single feed forward networks are employed and hybrid approaches where deep neural networks are themselves embedded in the iterative optimization process of solving the inverse problem. In principle, both approaches can be applied to EPT. Using the feedforward approach, Mandija et al. [94] employing convolutional neural networks demonstrated that deep learning EPT (DL-EPT) can reconstruct more noise robust dielectric parameter maps than conventional Helmholtz based EPT. An essential element of this feed forward approach is that the network constitutes a surrogate EPT reconstruction model implicitly learnt from the training data and takes the measured complex transmit field information as the input. This learning-based approach creates more flexibility than state-of-the-art MR-EPT techniques, which require electromagnetic quantities dictated by electromagnetic first principles which are not accessible with MRI. As example, in DL-EPT, a feedforward network can be trained with MR accessible quantities (e.g., the transceive phase) only. Interestingly, Mandija et al. [94] demonstrated that, also for a deep learning approach, almost all predictive features to reconstruct electrical conductivity are contained in the transceive phase maps in accordance with our insight from electromagnetic principles underpinning conventional EPT.

### 6.3. Training Data and Generalization to Unseen Data

Essential of course is the availability of training data which can nowadays be easily generated by electromagnetic simulations including realistic RF coil models, phantoms and body models. In this way, a high degree of a priori knowledge, such as the specific MRI coil setup, can be introduced. The advantage of using this simulation-based approach to generate the training transmit field data with superimposed artificial noise, is that the ground truth is available. However, in silica training data might not reflect realistic experimental conditions. Therefore, approaches that employ training data reconstructed with more conventional EPT reconstruction schemes are also used [95]. In the work of Gavazzi et al. [96], a 3D patch neural network approach was used where the receptive field is more local (size of the 3D patch) forcing it to perform dielectric parameter estimation from more local ${\hat{B}}_{1}^{+}$ magnitude and phase information. A further advantage is that it can work with varying matrix size of the input data. A key question is of course how a single feed forward neural network approach behaves when it is tested on unseen input data that was not directly included in the training data, e.g., pathologic tissue with different dielectric parameter values or in the presence of motion artifacts on the ${\hat{B}}_{1}^{+}$ maps. An obvious mitigation is to augment the training data sufficiently (e.g., part of the transmit maps can be artificially corrupted with motion artifacts) to obtain more robust results.

### 6.4. Deep Learning EPT: Integrating Deep Learning into Iterative EPT Schemes

Another option to improve the generalization to unseen data is to retain the physics in the reconstruction framework while still benefiting from the advantages of deep learning. A first approach was published for EPT by Leijsen et al. [97], who demonstrated that initial estimates provided by deep learning led to better convergence for 3D CSI-EPT. This integration can be further improved. New hybrid approaches are now emerging in medical image construction where neural networks are embedded in conventional iterative reconstruction schemes [98,99,100,101,102]. The physics related to the reconstruction problem is still explicitly included by means of a physics-based forward operator (e.g., Fourier transform in case of MR image reconstruction). Experiences from the medical image reconstruction demonstrated that this leads to much better generalization to unseen data in the training [100]. The neural networks can be inserted in the iterative procedure for various tasks. For example, neural networks can be trained to learn regularization filters much more tailored to the specific application than applying standard regularization kernels [101]. Alternatively, the networks can be used to perform the update task, i.e., determining the update direction based on the data mismatch and the regularization term [98]. Employing such an approach, the convergence is often much faster as a priori information on the optimization landscape is learned in the training phase, enabling faster convergence. These hybrid approaches should also be possible to combine with iterative EPT schemes such as 3D CSI-EPT where the physics is included by a forward operator (e.g., Green’s function approach) linking a certain electrical property distribution to the measured data (${\hat{B}}_{1}^{+}$ magnitude and transceive phase information). Such a methodology would be an ideal scenario as it would harvest the power of deep learning to accelerate reconstruction and include tailored a priori information from the learning phase, while still retaining to the physics-based modeling and the data consistency.

### 6.5. Outlook

It is clear that deep learning offers much benefit for EPT in terms of achieving higher quality reconstructions. The feedforward approaches using CNNs have demonstrated clear potential in terms of noise robustness, flexibility on inputs and computational speed. A key question is the generalization to data not encountered in the training. Interestingly, also in EPT’s sister field of Quantitative Susceptibility Mapping deep learning improves the quality of susceptibility reconstruction over conventional methodology [103,104]. Promising results have been achieved here on the generalization issue. An attractive alternative might be the integration of learned networks into a conventional iterative EPT approach as occurs in medical image reconstruction. This still retains the physics, offering better generalization, while also being able to include more a priori information and providing faster reconstructions.

## 7. Discussion and Conclusions

This paper presents a mathematical analysis of a large number of different methods for EPT, each with their own relative strengths and weaknesses. By comparing the results from each approach, one can make a number of general statements, the most important of which are listed below.

### 7.1. Approach Description

EPT approaches can be sorted into several different categories. These categories can give some general insight into how the methods work, and what kind of restrictions exist. Here, we sort the methods into three categories.

- *Differential methods* or *integral methods*
- *Local methods* that reconstruct the EPs at a specific location by only taking the information from the direct neighbourhood into account, or *global methods* that take the whole imaging domain into account to reconstruct the EP maps as a whole
- *Direct methods* that act directly on the data to reconstruct the EPs, also called backward methods since they run ‘backwards’ from the measured field map to the underlying EPs, or *forward methods* that employ forward models or solve forward problems in the inversion scheme and act ‘indirectly’ on the data

For each of the transmit field-based methods discussed in the manuscript, the approach descriptions are assigned in Table 1.

#### 7.1.1. Differential vs. Integral

**Figure 2:**
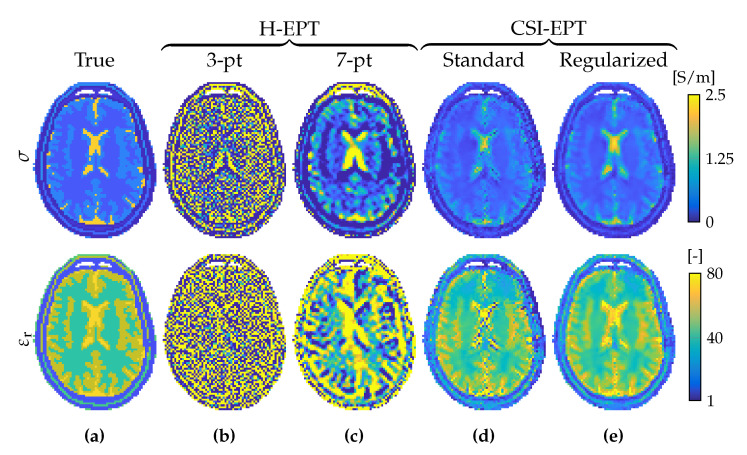
Direct differential method and forward integral method comparison on simulated three-dimensional noisy data (SNR=100) from a 7 T head coil. Methods are three-dimensional implementations without and with noise suppression in the form of using a larger differential kernel [32] or by including multiplicative total variation regularization [73]. True model (**a**), Helmholtz-based EPT with a 3-point kernel (**b**), Helmholtz-based EPT with a 7-point kernel (**c**), standard contrast source inversion EPT (**d**) and regularized contrast source inversion EPT (**e**). Conductivity (top row) and relative permittivity (bottom row).

A general observation is that differential approaches have an inherent noise amplification, while integral approaches are more noise robust due to the inherent low-pass filtering properties of the relevant integrals. The higher is the order of the differentials acting on the data, the larger is the noise amplification. A comparison between a second-order differential approach and an integral approach is shown for simulated three-dimensional noisy data (SNR = 100, as defined in [94]) in Figure 2. It shows that integral methods are in essence more noise robust and that typical noise reduction implementations such as regularization do not overcome this disadvantage.

#### 7.1.2. Local vs. Global

**Figure 3:**
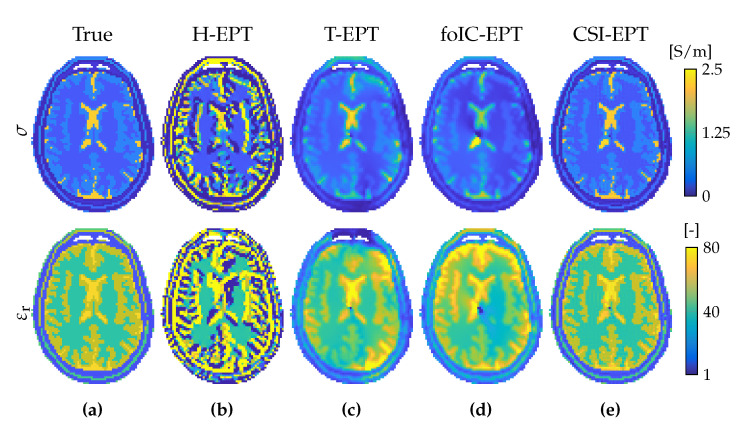
Local method and global methods comparison on simulated two-dimensional noiseless data from a 7 T head coil. Methods are two-dimensional implementations. True model (**a**), Helmholtz-based EPT (**b**), Transverse EPT (**c**), first-order Induced-Current EPT (**d**) and contrast source inversion EPT (**e**). Conductivity (top row) and relative permittivity (bottom row).

A commonality among many approaches is that there appears to be a trade-off between having an adverse noise effect in local methods due to higher-order derivatives (second order and up) acting on the data or having the bias effect of the EM field structure in global methods. A comparison between two-dimensional implementations of local and global approaches that assume knowledge of the complex transmit signal is shown for simulated two-dimensional noiseless data in Figure 3. The reconstructions of the local method H-EPT shows boundary errors due to assumed homogeneity of the underlying tissue, but the method is accurate in regions which have locally spatially invariant tissue properties. The reconstructions of the global methods (T-EPT, foIC-EPT and CSI-EPT) take the inhomogeneity of the EPs into account, but suffer from a bias related to the low electric field strength (low convective field). Additionally, global methods have the potential to reach local minima in their optimization process.

Global methods allow for the inclusion of regularization in the optimization problem which can be employed to correct for the bias, resolve local minima or improve noise robustness.

#### 7.1.3. Direct vs. Forward

A general observation is that direct methods are relatively fast, while forward methods tend to be computational expensive and time consuming, especially those that require the results of forward and/or inverse problems iteratively. Forward methods have yet to be demonstrated to be clinically feasible. Forward methods, however, typically simultaneously reconstruct additional field maps, such as the electric field strength which would be useful for SAR computations.

### 7.2. Data Requirements

EPT approaches can also be categorized on which type of data they require, or what kind of assumptions about the data are required. For each of the transmit field-based methods discussed in the manuscript, data requirements are assigned in Table 2.

#### 7.2.1. Measurable and Non-Measurable Data

For accurate reconstruction of the EPs, ideally measurements of all three components of the $B_{1}$ field would be possible. However, the *z*-component cannot be measured. Additionally, the *x*- and *y*-components cannot be acquired in a direct fashion, and determination would require the absolute transmit field as well as the absolute receive field. The absolute magnitude of the transmit field can be acquired in a direct fashion, while the absolute transmit phase and absolute receive field can not. Measured phases are always a superposition of the transmit and receive phase, and the magnitude of the receive field is always weighted by the proton-density. This data unavailability is one of the fundamental challenges that makes EPT complicated.

EPT approaches that assume availability of the complex transmit field typically estimate the absolute transmit phase by applying the transceive phase estimation (TPA), while methods that incorporate absolute receive fields go hand-in-hand with homogeneity or symmetry assumptions to eliminate the proton-density bias. To bypass these assumptions, solutions are often sought by reformulating the problem in terms of only directly-available field quantities. With regular RF coils, this comes down to formulating the inverse problem in only magnitude data or (in combination with) transceive phase data. However, with multi-element RF coil arrays, the acquisition of relative fields are possible, by dividing the complex signal measured in an element by the signal obtained in a particular reference element. This allows the derivation of the (gradient of the) transmit (and receive) phase, as well as EPT formulations based on the relative phase, instead of absolute or transceive phase, or formulations based on receive fields only. Additionally, this type of coil can eliminate specific artifacts since common terms for the different channels can be eliminated. However, the acquisition of multiple ${\hat{B}}_{1}^{+}$ fields requires lengthy scans which can compromise patient comfort, throughput or SNR. Moreover, these multi-element RF coil elements are not yet widely available in clinical settings. About 50% of clinical MR scanners have a field strength of 1.5 T and have a body coil with a single transmit channel. About 45% of clinical MR scanners have a field strength of 3 T, of which the older ones have the same arrangement, and the newer one typically have two independent transmit channels that can produce different degrees of elliptically polarized RF fields. High field scanners, such as 7 T scanners, can have up to eight transmit channels with independent magnitude and phase control; however, there are only about 100 of these worldwide available.

Integral methods often require knowledge of the incident electric and magnetic field strength which are inaccessible with MRI. Typically, a reference scan from a phantom with known EPs or a simulation setup is used for estimation of the incident fields. However, since the incident fields are dependent on the loading of the coil, they are to a certain level patient-specific. Patient-specific coil–subject interactions remain unknown and a source of error.

#### 7.2.2. Field/Object Structure

The inverse problem in EPT can be significantly simplified by assuming local homogeneity of the object. Methods that apply this local homogeneity assumption (LHA) are most often used in clinical studies due to their simplicity and ease of implementation. These methods however suffer from significant errors at tissue boundaries where the LHA is violated, making them impractical in regions with small tissue structures. For larger tissue structures, tissue segmentation can be used to improve boundary reconstructions.

The EPT problem can also be simplified by assuming an E-polarized field structure, i.e., assuming negligible (gradients of the) longitudinal component of the magnetic flux density, sometimes in combination with the assumption of vanishing (gradients of the) transverse components of the electric fields strength. This approximation is typically applied in the transverse midplane of a birdcage coil, where they are relatively small [13].

### 7.3. State Of Development

One might select a method based on different criteria, for example based on the SNR level, on the availability of multiple transmit or receive elements or incident fields, or whether or not the region of interest contains a homogeneous medium, a low electric field region or E-polarized fields. Due to the large number of EPT approaches with a large variety in requirements, assumptions and complexity, and since the EPT field is relatively new, most methods are not at the stage of clinical use yet (see Table 3). To facilitate development, comparison and prototyping of EPT approaches, MATLAB code of the approaches presented in Figure 2 and Figure 3 is available upon request from the corresponding author.

## Figures and Tables

**Table 1:** Approach description for the EPT approaches as described in this manuscript.

	H-EPT	SH-EPT	LMT	MDE-EPT	G-EPT	CR-EPT	T-EPT	foIC-EPT	VBIM-EPT	GMT	CSI-EPT	SA-EPT	I-EPT
Differential (order)	✓(2)	✓(2)	✓(2)	✓(2)	✓(2)	✓(2)	✓(1)	✓(1)	✗	✗	✗	✓(3)	✓(2)
Integral	✗	✗	✗	✓	✗	✗	✗	✓	✓	✓	✓	✗	✗
Local	✓	✓	✓	✓	✓	✗	✓	✓	✗	✗	✗	✓	✓
Global	✗	✗	✗	✗	✓	✓	✓	✓	✓	✓	✓	✗	✗
Direct (backward)	✓	✓	✓	✓	✓	✓	✓	✓	✗	✗	✗	✓	✓
Forward (indirect)	✗	✗	✗	✗	✗	✗	✓	✓	✓	✓	✓	✗	✗

**Table 2:** Data requirements for the EPT approaches as described in this manuscript. Note that data requirements can be influenced by extensions or generalizations of the methods.

	H-EPT	SH-EPT	LMT	MDE-EPT	G-EPT	CR-EPT	T-EPT	foIC-EPT	VBIM-EPT	GMT	CSI-EPT	SA-EPT	I-EPT
${\hat{B}}_{1}$ term	${\hat{B}}_{1}^{+}$	$\left|{\hat{B}}_{1}^{+}\right|,{\hat{\varphi }}^{+}$	$\left|{\hat{B}}_{1}^{+}\right|,{\hat{\varphi }}_{pq}^{\pm }$	${\hat{B}}_{1}^{+}$	$\left|{\hat{B}}_{1}^{+}\right|,{\hat{\varphi }}_{pr}^{+}$	${\hat{B}}_{1}^{+}$	${\hat{B}}_{1}^{+}$	${\hat{B}}_{1}^{+}$	${\hat{B}}_{1}^{+}$	${\hat{B}}_{1}^{+}$	${\hat{B}}_{1}^{+}$	$\left|{\hat{B}}_{1}^{-}\right|,{\hat{\varphi }}_{pq}^{-}$	${\hat{B}}_{1}^{+}{\hat{B}}_{1}^{-;*}$
Multi-element array	✗	✗	✓	✓	✓	✗	✗	✗	✗	✗	✗	✓	✗
Incident fields	✗	✗	✗	✗	✗	✗	✗	✓	✓	✓	✓	✗	✗
Seed Points	✗	✗	✗	✗	✓	✗	✗	✗	✗	✗	✗	✗	✗
∇η=0	✓	✓	✓	✗	✗	✗	✗	✗	✗	✗	✗	✓	✓
$\nabla \left|{\hat{B}}_{1}^{+}\right|=0$	✗	✓	✗	✗	✗	✗	✗	✗	✗	✗	✗	✗	✗
$\nabla {\hat{\varphi }}^{+}=0$	✗	✓	✗	✗	✗	✗	✗	✗	✗	✗	✗	✗	✗
$\partial_{x}{\hat{B}}_{z}=0$	✗	✗	✗	✓	✓	✓	✗	✗	✗	✗	✗	✗	✗
$\partial_{y}{\hat{B}}_{z}=0$	✗	✗	✗	✓	✓	✓	✗	✗	✗	✗	✗	✗	✗
$\partial_{z}{\hat{B}}_{z}=0$	✗	✗	✗	✓	✓	✓	✓	✓	✗	✗	✗	✗	✗
$\partial_{z}{\hat{E}}_{x}=0$	✗	✗	✗	✗	✗	✗	✓	✓	✗	✗	✗	✗	✗
$\partial_{z}{\hat{E}}_{y}=0$	✗	✗	✗	✗	✗	✗	✓	✓	✗	✗	✗	✗	✗
${\hat{B}}_{1}^{+}={\hat{B}}_{1}^{-;*}$	✗	✗	✗	✗	✗	✗	✗	✗	✗	✗	✗	✗	✓

**Table 3:** Type of experiments performed for the EPT approaches as described in this manuscript. The table is constructed to the best of the authors knowledge. For references, see the main text.

	H-EPT	SH-EPT	LMT	MDE-EPT	G-EPT	CR-EPT	T-EPT	foIC-EPT	VBIM-EPT	GMT	CSI-EPT	SA-EPT	I-EPT
Simulation	✓	✓	✓	✓	✓	✓	✓	✓	✓	✓	✓	✓	✓
Phantom	✓	✓	✗	✗	✓	✓	✗	✓	✗	✓	✓	✓	✓
in vivo	✓	✓	✗	✗	✓	✗	✗	✗	✗	✗	✗	✗	✓
Clinical	✗	✓	✗	✗	✗	✗	✗	✗	✗	✗	✗	✗	✗

## Abbreviations

The following abbreviations are used in this manuscript:

CR-EPT	Convection–reaction EPT
CSI-EPT	Contrast source inversion EPT
DL-EPT	Deep-learning EPT
ECG	Electrocardiography
EEG	Electroencephalography
EM	Electromagnetic
EP	Electrical property
EPT	Electrical properties tomography
foIC-EPT	First-order induced-current EPT
G-EPT	Gradient-based EPT
GMT	Global Maxwell tomography
H-EPT	Helmholtz-based EPT
I-EPT	Image-based EPT
LMT	Local Maxwell tomography
MDE-EPT	Modified dual-excitation EPT
MR	Magnetic resonance
MRI	Magnetic resonance imaging
P-CM	Poisson-based conductivity mapping
PCR-CM	Phase-only convection–reaction conductivity mapping
RF	Radio Frequency
SA-EPT	Single-acquisition EPT
SH-EPT	Simplified H-EPT
SNR	Signal-to-noise ratio
T-EPT	Transverse EPT
VBIM-EPT	Variational Born iterative method EPT
